# CONSORT-EHEALTH: Improving and Standardizing Evaluation Reports of Web-based and Mobile Health Interventions

**DOI:** 10.2196/jmir.1923

**Published:** 2011-12-31

**Authors:** Gunther Eysenbach, CONSORT-EHEALTH Group

**Affiliations:** ^1^University Health NetworkCentre for Global eHealth Innovation & Techna InstituteToronto, ONCanada; ^2^Institute for Health Policy, Management, and EvaluationUniversity of TorontoToronto, ONCanada; ^3^JMIR Publications Inc.Toronto, ONCanada; ^4^see Acknowledgments for contributors

**Keywords:** evaluation, Internet, mobile health, reporting standards, publishing standards, guidelines, quality control, randomized controlled trials as topic, medical informatics

## Abstract

**Background:**

Web-based and mobile health interventions (also called “Internet interventions” or "eHealth/mHealth interventions") are tools or treatments, typically behaviorally based, that are operationalized and transformed for delivery via the Internet or mobile platforms. These include electronic tools for patients, informal caregivers, healthy consumers, and health care providers. The Consolidated Standards of Reporting Trials (CONSORT) statement was developed to improve the suboptimal reporting of randomized controlled trials (RCTs). While the CONSORT statement can be applied to provide broad guidance on how eHealth and mHealth trials should be reported, RCTs of web-based interventions pose very specific issues and challenges, in particular related to reporting sufficient details of the intervention to allow replication and theory-building.

**Objective:**

To develop a checklist, dubbed CONSORT-EHEALTH (Consolidated Standards of Reporting Trials of Electronic and Mobile HEalth Applications and onLine TeleHealth), as an extension of the CONSORT statement that provides guidance for authors of eHealth and mHealth interventions.

**Methods:**

A literature review was conducted, followed by a survey among eHealth experts and a workshop.

**Results:**

A checklist instrument was constructed as an extension of the CONSORT statement. The instrument has been adopted by the *Journal of Medical Internet Research* (JMIR) and authors of eHealth RCTs are required to submit an electronic checklist explaining how they addressed each subitem.

**Conclusions:**

CONSORT-EHEALTH has the potential to improve reporting and provides a basis for evaluating the validity and applicability of eHealth trials. Subitems describing how the intervention should be reported can also be used for non-RCT evaluation reports. As part of the development process, an evaluation component is essential; therefore, feedback from authors will be solicited, and a before-after study will evaluate whether reporting has been improved.

## Introduction

### Introducing CONSORT-EHEALTH

The current issue of the *Journal of Medical Internet Research* (JMIR) 4/2011 is the first issue where we asked JMIR authors describing randomized trials to report their trials in accordance with a new instrument designed to improve the quality of reporting of eHealth and mHealth trials, dubbed CONSORT-EHEALTH (Consolidated Standards of Reporting Trials of Electronic and Mobile HEalth Applications and onLine TeleHealth). While completing the checklist is only mandatory for authors of randomized controlled trials (RCTs), the checklist may also be useful for researchers employing other evaluation methods. Beyond web-based and mobile applications, the checklist presented here is probably also applicable for a wide range of other medical informatics and technology applications. This editorial provides a short introduction on the rationale and background of this ongoing initiative to improve the quality of research and to improve knowledge translation in this field. 

### Web-based and Mobile Health Interventions

Web-based health interventions (also called “Internet interventions” or “eHealth interventions”) are, for the purpose of this paper, “treatments, typically behaviorally based, that are operationalized and transformed for delivery via the Internet” [1]. With mobile devices being an increasingly important access point for Internet-based or otherwise networked electronic interventions, this definition includes interventions that are delivered through mobile devices or the new generation of tablet computers (eg, the iPad). Examples are behavior change interventions that help people quit smoking or lose weight, or mental health applications to address depression, anxieties, or other important health problems. An increasingly important area is the management of chronic diseases such as diabetes using Internet-based or mobile disease management programs, as well as patient-accessible personal health records, and tailored educational programs for patients. The term “treatment” should be understood in its broadest sense, and includes, for example, management tools, electronic tools that improve the communication (eg, between patient or health professionals) or systems that provide diagnostic or prognostic information or aid in the triage of patients. 

Web-based and mobile interventions are increasingly important instruments in the toolkit of public health professionals and researchers [1-3]. The web-based delivery mode makes it relatively easy to enroll and track a large number of participants in longitudinal studies, including RCTs, to test the effectiveness of specific program components, or to evaluate the effectiveness of the program as a whole. The ease of enrollment comes, however, at a cost: compared to face-to-face trials, researchers in eHealth trials have less control over the participants, and Internet-based trials pose some other specific problems, related to execution and reporting of the trial [4].

While this is a young field, with less than a dozen web-based RCTs published before 2002 [4, 5], the number of reports evaluating web-based interventions in the medical literature is increasing rapidly. In October 2010, a scan of literature indexed in PubMed with the publication type “randomized trial” and major medical subject headings (MeSH) term “Internet”, elicited 582 published randomized trials. This does not take into account evaluations of mobile networked applications (which may not be indexed with the “Internet” keyword), or studies with nonrandomized longitudinal designs.

In addition to the rapidly growing area of Internet interventions, RCTs evaluating non-Internet interventions are also using elements of web-based trials, for example, Web-based recruitment or Web-based data collection [6].

While JMIR is the leading journal in this field (in terms of both impact and number of articles published in this field), these trials are scattered across a wide variety of journals: in the October 2010 scan, 263 different journals were identified which have published at least one “eHealth RCT”. While JMIR was the journal which had most trials published, the distribution has a very long tail, with relevant articles scattered in a wide range of other journals (see Table 1). As a consequence, reporting standards and the level of detail provided in these publications vary widely, hampering progress in this area, and impeding knowledge translation. While at JMIR we are requiring authors to submit the CONSORT (Consolidated Standards of Reporting Trials) checklist [7-11] and use additional checklists for some aspects of these trials (eg, Checklist for Reporting Results of Internet E-Surveys [CHERRIES] [12]), internationally developed and adopted reporting guidelines specifically for eHealth and mHealth trials are lacking.

The CONSORT statement was developed to improve the suboptimal reporting of RCTs [9]. While the CONSORT statement [9] and some published extensions [7,8,10,11] as well as other guidelines for other study designs and domains can be applied to provide broad guidance on how such evaluations should be reported, RCTs of web-based interventions pose very specific issues and challenges [4, 13], which we intended to amalgamate and elaborate in the form of a eHealth-specific CONSORT extension guideline and checklist, called CONSORT-EHEALTH. 

**Table 1:** Ranking of journals which have published at least 4 randomized trials of web-based applications (indexed with “Internet” as major MeSH heading and publication type = RCT) [from a list of a total of 582 trial publications], as of October 2010 (journal titles as per PubMed)

Journal name	number of Internet RCTs
Journal of medical Internet research (JMIR)	37
Preventive medicine	12
Journal of consulting and clinical psychology	11
Nicotine & tobacco research: official journal of the Society for Research on Nicotine and Tobacco	10
Diabetes care	9
Health education research	9
Behaviour research and therapy	9
Cyberpsychology & behavior: the impact of the Internet, multimedia and virtual reality on behavior and society	9
Academic medicine: journal of the Association of American Medical Colleges	8
Journal of health communication	8
Cognitive behavior therapy	8
The Australian and New Zealand journal of psychiatry	7
BMC psychiatry	7
Studies in health technology and informatics	7
Annals of behavioral medicine: a publication of the Society of Behavioral Medicine	7
Pediatrics	6
Patient education and counseling	6
Addiction (Abingdon, England)	6
Journal of substance abuse treatment	6
American journal of preventive medicine	6
BMC medical education	5
Obesity (Silver Spring, Md.)	5
Behavior research methods, instruments, & computers: a journal of the Psychonomic Society, Inc	5
Archives of internal medicine	5
Addictive behaviors	5
Journal of nutrition education and behavior	5
Proceedings / AMIA ... Annual Symposium. AMIA Symposium	5
The Journal of adolescent health: official publication of the Society for Adolescent Medicine	5
BMC public health	5
AMIA ... Annual Symposium proceedings / AMIA Symposium. AMIA Symposium	5
Journal of general internal medicine	5
Trials	5
JAMA: the journal of the American Medical Association	4
The British journal of psychiatry: the journal of mental science	4
Journal of pediatric psychology	4
Journal of clinical nursing	4
Health psychology: official journal of the Division of Health Psychology, American Psychological Association	4
Computers, informatics, nursing: CIN	4
Depression and anxiety	4
Journal of telemedicine and telecare	4

## Methods

To develop a guideline for reports of web-based interventions we broadly followed the standard methodology developed by the CONSORT group, reported in detail elsewhere [14]. We started the work on CONSORT-EHEALTH in October 2010 with writing a grant proposal requesting funding for a consensus workshop from the Canadian Institutes of Health Research (CIHR). Unfortunately, this funding request was turned down (with some rather odd explanations, such as “[it is unclear] why journal editors [private sector] need funding to complete this project.”) Without funding, our initial plan to use a 3-phase process of premeeting item generation, a meeting with invited stakeholders, and postmeeting consolidation, had to be modified, with only a very short face-to-face workshop in the context of a scientific meeting, and the bulk of the work being conducted through online consultations.

The core international group of CONSORT-EHEALTH contributors included researchers, funders, consumers, journal editors, and industry, listed under acknowledgments. This is (and remains) an open and dynamic group.

In the premeeting item-generation process, we used the current CONSORT guideline items as a framework, and generated additional items and subitems through literature searches, extracting reported items from published RCTs as well as relevant guidelines. We had access to the referee reports of JMIR, which helped us to evaluate which items are frequently pointed out by reviewers as “missing” in the original submissions of the authors. Additional input came from a face-to-face session hosted by the International Society for Research on Internet Interventions (ISRII), in Sydney, Australia on April 6-8, 2011.

A preliminary version of the CONSORT-EHEALTH checklist (V1.5) was published in April 2010. In a web-based Delphi process we gathered some data on the importance of the items [14]. The initial questionnaire with the list of items is shown in Multimedia Appendix 1. Participants were asked to suggest missing items (under each CONSORT subheading), and to rate each proposed subitem on a scale of 1-5 (where 1 was “subitem not at all important” and 5 was “essential”).

We kept items as “essential” in the CONSORT-EHEALTH when at least 50% of respondents rated an item as “5-essential”. We downgraded items as “highly recommended” when at least 50% of respondents rated an item as 4 or 5 (but less than 50% said it is “essential”). We eliminated items when less than 50% of respondents answered 4 or 5.

## Results

### CONSORT-EHEALTH

The key references identified and used for item generation are the original CONSORT items–in particular including the extension for nonpharmacologic treatments [7]–as well as an early paper by Eysenbach on eHealth-specific RCT issues [4], a paper on the relevance of CONSORT reporting criteria for eHealth trials by Baker and colleagues [13], and a preprint of a recent paper by Proudfoot and colleagues [15]. An existing guideline for evaluation studies in health informatics [16] proved to be too broad to be of much use. Systematic reviews in this area [2, 3, 17, 18] provided further useful frameworks for identifying items that should be reported.

Based on these references, we published an initial instrument (CONSORT-EHEALTH V1.5) with 53 additional subitems, either explaining or enhancing the original 25 CONSORT items. EHEALTH-specific subitems were indicated with Roman numerals (eg, CONSORT item 2a had two additional subitems numbered 2a-i and 2a-ii). We added 2 items to the original 25-item CONSORT (item X26 on ethics, and item X27 on conflict of interest disclosure), which are not part of the original CONSORT checklist and did not fit under any existing item. This instrument was published in March 2011, and JMIR readers and eHealth stakeholders were asked to comment on the instrument and to rate the importance of each subitem.

Between April 4 and June 17 2011, we received 55 responses to CONSORT V1.5 (first Delphi round). Multimedia Appendix 2 and Multimedia Appendix 3 show the responses.

Most users agreed with all subitems. Only 1 subitem was eliminated after the first Delphi round (“Report how institutional affiliations are displayed to potential participants [on eHealth media], as affiliations with prestigious hospitals or universities may affect volunteer rates, use, and reactions with regard to an intervention”).

 The resulting current iteration of CONSORT-EHEALTH V1.6.1 (which is currently in use at JMIR) has 17 subitems that are deemed “essential”, and 35 subitems that are deemed “highly recommended” (Multimedia Appendix 4). The checklist (V1.6.1) was published on the JMIR website on August 25, 2011 and is currently being pilot-tested with the help of JMIR authors, who are asked to submit an electronic version of the checklist via an online questionnaire when they submit reports of an RCT (Multimedia Appendix 5). In this questionnaire, authors of RCTs are required to quote passages of their manuscript corresponding to each item, or to quickly explain why they are not applicable. They are also asked to (on a voluntary basis) rate the importance of the items for their trial. We expect the next iteration of CONSORT-EHEALTH (V2.0) to be published early 2013, which will take into account the feedback received from actual RCT authors (who may also be acknowledged as part of the CONSORT-EHEALTH revision group). This way we ensure that the next iteration of CONSORT-EHEALTH is informed by the experiences of actual users.

### Specific Reporting Issues of Ehealth Trials and Examples for Guideline Items

In the following we provide some examples of items and subitems that are part of the guideline. 

For example, in the interest of reproducibility and comparability (in the research setting) as well as for implementation and dissemination (in practice settings), a detailed description and documentation of the intervention is required. The CONSORT checklist contains only a single item related to the intervention (item 5: “Describe the interventions for each group with sufficient details to allow replication, including how and when they were actually administered”). This may be sufficient for drug trials, where the simple mentioning of the drug name and its administration mode and dosage is sufficient to characterize the intervention, but not sufficient for eHealth or mHealth interventions, which are complex interventions requiring more details so that others can replicate the intervention. Thus, we created a detailed subchecklist as an extension to the CONSORT item 5, listing required and desired reporting elements characterizing the functional components and other important features of the web intervention (Table 2).

Two of these subitems (5-v and 5-vi) speak to the problem of digital preservation of the intervention, which is another unique aspect of eHealth or mHealth trials. For scientific hypotheses and findings to be confirmed or disproved by other researchers, key elements should be available to other researchers, ideally as open source code, or at least be theoretically “reproducible” by disclosing algorithms, pathways of participants through the application, etc., or at a minimum by providing screenshots or archiving the interfaces in a webarchive (such as the Internet Archive or WebCitation.org). The issue of open source and complete transparency of the algorithms appeared to be somewhat controversial among respondents, as some eHealth applications may have commercial use and some respondents were concerned about publicizing proprietary algorithms. While at JMIR we highly encourage the publication of open source code alongside the publication, there does not currently seem to be a consensus to make this a universal requirement across journals. However, even if the code is not made accessible as open source, the report must contain sufficient details and preferably screenshots to allow others to replicate or disprove the key findings – otherwise it cannot be considered scientific research and should be published in a trade journal rather than a peer-reviewed scholarly journal.

Apart from expanding the intervention description item 5, there were other important additions and expansions of the current CONSORT items.

A number of guideline subitems (6a-ii, 12a-i, 13b-i, 17-i; see Multimedia Appendix 4) are related to the important issue of attrition (non-use) and use (engagement, “dose”, adherence) of the intervention [19]. As participants in web-based evaluations usually have full control over whether or not they use the intervention, and how often and how long they engage with the application, real-world evaluations of web-based interventions and interpretations of reports on their effectiveness (or lack thereof) are often complicated by the fact that a substantial proportion of participants may have dropped out of the trial (non-use or loss-to-follow-up attrition) [19]. While nonadherence may be a problem in drug trials too, the attrition rates in Internet-based trials are by far higher than in trials with a face-to-face component. As effectiveness as measured in these trials is a function of (and dependent on) participants actually using the intervention, researchers should measure and report metrics of use (adherence) and/or non-use (attrition), which can be measured using a variety of metrics such as number of logins and average session time. However, even these seemingly straightforward metrics require additional explanations, for example, if researchers report an average session time, this may be skewed by some participants never logging out; therefore, additional information such as the timeout policy should be provided (eg, automatic logout after 15 minutes of inactivity) in order to enable accurate interpretation and across-trial comparisons. In subitem 6a-ii (an expansion of CONSORT item 6 “outcomes”) we suggest that researchers explain how use and engagement was measured and defined, in addition to describing how the primary health outcomes were measured, and in subitem 17-i (an expansion of CONSORT item 17 “outcomes and estimation”) we ask that use and usage outcomes should be reported. In subitem 12a-i (an expansion of CONSORT item 12 “statistical methods”) we specifically ask how missing values due to attrition were treated statistically [20]. In addition to the traditional CONSORT flow diagram we also highly encourage the provision of an attrition diagram (CONSORT-EHEALTH item 13b-i) in the results sections, illustrating the login behavior of participants in all groups over time as a survival curve [19].

The comprehensive description of web-based recruitment strategies and data collection methods are other areas where we identified the need for guideline items. Our previously published CHERRIES guideline for reporting web-based surveys [12] may provide additional guidance and may be seen as a supplement to subitem 6a-i, which deals with the common case where outcomes were collected through online questionnaires.

There is a regrettable trend to split reports of randomized trials into “least publishable units”, for example, to publish one paper with the results of the primary RCT outcomes, another paper with usage results, and another paper with a qualitative analysis of participant feedback. At JMIR, we have a strict policy against “salami publication”, a practice that limits the ability of the reader to interpret the overall findings, and will consider such multipart papers only in exceptional circumstances, and preferably when the reports are submitted together and published in the same journal issue. An in-depth qualitative evaluation may justify a separate paper, but a few CONSORT-EHEALTH items (6a-iii and 19-ii) remind authors that some qualitative analysis should be part of any eHealth evaluation report, in particular if nonuse of the application or potential harmful effects were observed, which should shift the focus of the report to the question *why* these results occurred.

Finally, in order to enhance retrievability (findability) of these kinds of studies in PubMed and other bibliographic databases, we also suggest preferred terms to be used in article titles and abstracts (e.g. “web-based intervention” or “mobile intervention”). These recommendations are based on an analysis of the prevalence of terms used in current studies.

**Table 2:** Subitems expanding CONSORT item 5 (description of intervention)

	Subitem	Importance
i)	**Mention names, credential, affiliations of the developers, sponsors, and owners** [15] (if authors/evaluators are owners or developer of the software, this needs to be declared in a “Conflict of interest” section).	Highly Recommended
ii)	**Describe the history/development process** of the application and previous formative evaluations (e.g., focus groups, usability testing), as these will have an impact on adoption/use rates and help with interpreting results.	Highly Recommended
iii)	**Revisions and updating**. Clearly mention the date and/or version number of the application/intervention (and comparator, if applicable) evaluated, or describe whether the intervention underwent major changes during the evaluation process, or whether the development and/or content was “frozen” during the trial. Describe dynamic components such as news feeds or changing content which may have an impact on the replicability of the intervention (for unexpected events see item 3b).	Highly Recommended
iv)	Provide information on **quality assurance methods** to ensure accuracy and quality of information provided [13], if applicable.	Highly Recommended
v)	**Ensure replicability by publishing the source code (preferably as open source), and/or providing screenshots/screen-capture video, and/or providing flowcharts of the algorithms used.** Replicability (i.e., other researchers should in principle be able to replicate the study) is a hallmark of scientific reporting.	Highly Recommended
vi)	**Digital preservation**: Provide the URL of the application, but as the intervention is likely to change or disappear over the course of the years, also make sure the intervention is archived (Internet Archive, webcitation.org, and/or publishing the source code or screenshots/videos alongside the article). As pages behind login screens cannot be archived, consider creating demo pages which are accessible without login.	Highly Recommended
vii)	**Access**: Describe how participants accessed the application, in what setting/context, if they had to pay (or were paid) or not, whether they had to be a member of specific group. If known, describe how participants obtained “access to the platform and Internet” [13]. To ensure access for editors/reviewers/readers, consider providing a “backdoor” login account or demo mode for reviewers/readers to explore the application (also important for archiving purposes, see vi).	Essential
viii)	Describe **mode of delivery, features/functionalities/components of the intervention and comparator, and the theoretical framework** [6] used to design them (instructional strategy [13], behavior change techniques, persuasive features, etc., see e.g., [17, 18] for terminology). This includes an in-depth description of the content (including where it is coming from and who developed it) [13], “whether [and how] it is tailored to individual circumstances and allows users to track their progress and receive feedback” [15]. This also includes a description of communication delivery channels and – if computer-mediated communication is a component – whether communication was synchronous or asynchronous [15]. It also includes information on presentation strategies [13], including page design principles, average amount of text on pages, presence of hyperlinks to other resources etc. [13].	Essential
ix)	**Describe use parameters** (e.g., intended “doses” and optimal timing for use) [13]. Clarify what instructions or recommendations were given to the user, for example, regarding timing, frequency, heaviness of use [13], if any, or was the intervention used ad libitum.	Highly Recommended
x)	**Clarify the level of human involvement** (care providers or health professionals, also technical assistance) in the e-intervention or as co-intervention. Detail number and expertise of professionals involved, if any, as well as “type of assistance offered, the timing and frequency of the support, how it is initiated, and the medium by which the assistance is delivered” [15]. It may be necessary to distinguish between the level of human involvement required for the trial, and the level of human involvement required for a routine application outside of an RCT setting (discuss under item 21 – generalizability).	Highly Recommended
xi)	**Report any prompts/reminders used:** Clarify if there were prompts (letters, emails, phone calls, SMS) to use the application, what triggered them, frequency, etc. [13]. It may be necessary to distinguish between the level of prompts/reminders required for the trial, and the level of prompts/reminders for a routine application outside of an RCT setting (discuss under item 21 – generalizability).	Essential
xii)	**Describe any co-interventions (including training/support)**: Clearly state any “interventions that are provided in addition to the targeted eHealth intervention” [13], as eHealth intervention may not be designed as stand-alone intervention. This includes training sessions and support [13]. It may be necessary to distinguish between the level of training required for the trial, and the level of training for a routine application outside of an RCT setting (discuss under item 21 – generalizability).	Essential

## Discussion

We hypothesize that publication of the guideline in August 2010 will have a significant impact on the quality of reports of web-based intervention evaluations, which will in turn enable better systematic reviews and facilitate knowledge translation. The guideline will hopefully also be a useful starting point and framework for discussions around the quality of eHealth trials, how such trials should actually be conducted, which items should be reported in protocols, grant proposals and trial registries, and how trials should be classified and synthesized in systematic reviews.

Elements of the guideline may be useful for researchers of other disciplines who use web-based recruitment or data collection methods, even if it is not an Internet- or mobile intervention which is being evaluated.

Many elements of the guideline (particularly the section describing subitems of the intervention) are applicable not only to randomized trials, but any kind of evaluation report.

While the *Journal of Medical Internet Research* is the first journal to adopt these guidelines, we hope that other journals and organizations endorse and adopt the guidelines. Authors are encouraged to report their research (and research protocols) in accordance with CONSORT-EHEALTH, regardless of the ultimate publication venue. Authors preparing their reports in accordance with CONSORT-EHEALTH are encouraged to cite the current guidelines (this paper), in order to facilitate further dissemination and uptake of best practices for reporting.

The current checklist is only the first step and the guideline will be very much a living document in an iterative and ongoing development process. As technology is changing constantly and rapidly, and reporting of eHealth and mHealth interventions is determined by what is technologically possible, the checklist will need to be updated much more frequently than other guidelines dealing with more “static” interventions, such as acupuncture (STRICTA) [21].

As part of the iterative development process, an ongoing evaluation component is essential; otherwise, asking authors, journals and editors to use and endorse the guidance is not warranted.

To provide a body of evidence to support usage of the guideline we intend to evaluate, elaborate on, and further develop the CONSORT-EHEALTH checklist, by

- a pilot implementation at the *Journal of Medical Internet Research* which involves collecting data from RCT authors (this pilot started with the current issue 4/2011) (see Multimedia Appendix 5 for the data collection form)
- a retrospective analysis of a random sample of web-based RCTs, published before publication of the CONSORT-EHEALTH checklist (ongoing)
  development of an Explanation and Elaboration manuscript
- development of a website and an interactive toolkit
- the formation of a standing working group to lead the continued development of the guideline
- a systematic analysis of RCTs of web-based interventions published after publication of the guideline, to evaluate the impact of the checklist, and to identify shortcomings and new items
- creation of a searchable database of trials (based on the information entered by JMIR authors when filling in the CONSORT-EHEALTH checklist).

It should be stressed again that the development of CONSORT-EHEALTH is an iterative and ongoing process, which requires a broad stakeholder input, which we welcome. We will continue to try to obtain funding for this important work which in our view is essential to advance the art and science of Internet and mobile interventions.

## Multimedia Appendix 1

Online questionnaire of the initial CONSORT-EHEALTH instrument V1.5.

## Multimedia Appendix 2

Summary of responses from the online questionnaire of CONSORT-EHEALTH V1.5 (note that narrative responses are excerpts only).

## Multimedia Appendix 3

Database with all responses received between April 4 and June 17, 2011, in response to the CONSORT-EHEALTH V1.5 questionnaire (email addresses and certain names removed).

## Multimedia Appendix 4

Current CONSORT-EHEALTH V1.6.1 checklist.

## Multimedia Appendix 5

Submission form for JMIR (based on CONSORT-EHEALTH V1.6), available at http://tinyurl.com/consort-ehealth-v1-6.
