# Supplementary material for: CONSORT-EHEALTH: Improving and Standardizing Evaluation Reports of Web-based and Mobile Health Interventions
Source: J Med Internet Res. 2011 Dec 31;13(4):e126. doi: 10.2196/jmir.1923 (PMC3278112; doi:10.2196/jmir.1923)
Supplement: Supplementary file 1 [file jmir_v13i4e126_app1.pdf]

CONSORT-EHEALTH (Draft V 1.5 2011-04-04) eDelphi Consensus Building ROUND 1

The CONSORT-EHEALTH checklist is intended for authors of randomized trials evaluating web-based and Internet-based applications/interventions, including mobile interventions, electronic games (incl multiplayer games), social media, and other interactive and/or networked electronic applications. Some of the items (e.g. all subitems under item 5 - description of the intervention) may also be applicable for other study designs.

The goal of the checklist is to be a) a guide for reporting for authors of RCTs, b) to form a basis for appraisal of an ehealth trial (in terms of validity)

CONSORT-EHEALTH items/subitems will be MANDATORY reporting items for studies published in the Journal of Medical Internet Research and other journals / scientific societies endorsing the checklist.

Items numbered 1., 2., 3., 4a., 4b etc are original CONSORT or CONSORT-NPT (non-pharmacologic treatment) items.
Items with Roman numerals (i., ii, iii, iv etc.) are proposed CONSORT-EHEALTH extensions/clarifications.
BELOW, PLEASE RATE THE IMPORTANCE OF THESE PROPOSED NEW SUB-ITEMS AND/OR COMMENT ON EACH ITEM. (comments could also include original references to be cited to support the subitem).

This is a Delphi survey to obtain feedback from ehealth and reporting guidelines experts (including journal editors).
If something important is missing which in your opinion must be part of EVERY reported ehealth trial, please add an item, but remember that there will be a separate Exploration&Elaboration document - the checklist should only contain essential and universally applicable items.

Subitems should be included in the new CONSORT-EHEALTH checklist if one of the following conditions for the subitem are met:
- if not conducted properly it may lead to empirical evidence of bias or threaten internal validity
- if not reported properly this is associated with empirical evidence of bias
- it may be associated with the success of the trial
- it may be associated with external validity (applicability or success of the application/intervention in other settings)
- it reflects crucial trial results
- it aids in the interpretation of results

At the same time, the CONSORT-EHEALTH checklist should be reasonably brief and universally applicable, so only essential items should be included.

* Required

Your name *
First Last

Primary Affiliation (short), City, Country *
University of Toronto, Toronto, Canada

Your e-mail address *
abc@google.com

Do you want to be acknowledged (with your name and affilitation) in the CONSORT-EHEALTH publication? *
(provided you make contributions below. Please refer to the last question if you also wish to be a co-author of the Elaboration manuscript)

- yes
- no

Your role/experience with ehealth-trials
Which of the following describes you (multiple may apply)

- I have experience with conducting ehealth studies myself, but no RCTs
- I have conducted ehealth RCTs
- I have read many ehealth RCT/evaluation reports
- I have experience mainly from a consumer/patient point of view
- I have experience mainly from a policy/implementation/decision-maker point of view

Other guidelines
Are you aware of any other guidelines that should be cited? (give references and provide a short description)

TITLE AND ABSTRACT

1a) Identification as a randomized trial in the title

1a-i) Identify the mode of delivery in the title

Identify the mode of delivery. Preferably use “web-based” and/or “mobile” and/or “electronic game” in the title. Avoid ambiguous terms like “online”, “virtual”, “interactive”. Use “Internet-based” only if Intervention includes non-web-based Internet components (e.g. email), use “computer-based” or “electronic” only if offline products are used. Use “virtual” only in the context of “virtual reality” (3-D worlds). Use “online” only in the context of “online support groups”. Complement or substitute product names with broader terms for the class of products (such as “mobile” or “smart phone” instead of “iphone”), especially if the application runs on different platforms.

	1	2	3	4	5	
subitem not at all important	○	○	○	○	○	essential

Comment on subitem 1a-i)

1a-ii) Non-web-based components or important co-interventions in title

Mention non-web-based components or important co-interventions in title, if any (e.g., “with telephone support”).

	1	2	3	4	5	
subitem not at all important	○	○	○	○	○	essential

Comment on subitem 1a-ii)

1a-iii) Primary condition or target group in the title

Mention primary condition or target group in the title, if any (e.g., “for children with Type I Diabetes”) Example: A Web-based and Mobile Intervention with Telephone Support for Children with Type I Diabetes: Randomized Controlled Trial

1 2 3 4 5

subitem not at all important ○ ○ ○ ○ ○ essential

Comment on subitem 1a-iii)

Add a subitem under CONSORT item 1a

1b) Structured summary of trial design, methods, results, and conclusions

NPT extension: Description of experimental treatment, comparator, care providers, centers, and blinding status.

1b-i) Key features/functionalities/components of the intervention and comparator in the abstract

Mention key features/functionalities/components of the intervention and comparator in the abstract. If possible, also mention theories and principles used for designing the site. Keep in mind the needs of systematic reviewers and indexers by including important synonyms.

	1	2	3	4	5	
subitem not at all important	○	○	○	○	○	essential

Comment on subitem 1b-i)

1b-ii) Level of human involvement in the abstract

Clarify the level of human involvement in the abstract, e.g., use phrases like “fully automated” vs. “therapist/nurse/care provider/physician-assisted” (mention number and expertise of providers involved, if any).

	1	2	3	4	5	
subitem not at all important	○	○	○	○	○	essential

Comment on subitem 1b-ii)

1b-iii) Open vs. closed, web-based (self-assessment) vs. face-to-face assessments in abstract

Open vs. closed, web-based (self-assessment) vs. face-to-face assessments in abstract: Mention how participants were recruited (online vs. offline), e.g., from an open access website (open trial) or from a clinic or other closed user group (closed trial), and clarify if this was a purely web-based trial, or there were face-to-face components (as part of the intervention or for assessment). Clearly say if outcomes were self-assessed through questionnaires (as common in web-based trials).

	1	2	3	4	5	
subitem not at all important	○	○	○	○	○	essential

Comment on subitem 1b-iii)

1b-iv) Results in abstract must contain use data

Report number of participants enrolled/assessed in each group, the use/uptake of the intervention (e.g., attrition/adherence metrics, use over time, number of logins etc.), in addition to primary/secondary outcomes.

	1	2	3	4	5	
subitem not at all important	○	○	○	○	○	essential

Comment on subitem 1b-iv)

1b-v) Conclusions/Discussions in abstract for negative trials

Conclusions/Discussions in abstract for negative trials: Discuss the primary outcome - if the trial is negative (primary outcome not changed), and the intervention was not used, discuss whether negative results are attributable to lack of uptake and discuss reasons.

	1	2	3	4	5	
subitem not at all important	○	○	○	○	○	essential

Comment on subitem 1b-v)

Add a subitem under CONSORT item 1b

INTRODUCTION

2a) Scientific background and explanation of rationale

2a-i) Problem and the type of system/solution

Describe the problem and the type of system/solution that is object of the study: intended as stand-alone intervention vs. incorporated in broader health care program? [1] Intended for a particular patient population? [1] Goals of the intervention, e.g., being more cost-effective to other interventions [1], replace or complement other solutions? (Note: Details about the intervention are provided in “Methods” under 5)

	1	2	3	4	5	
subitem not at all important	○	○	○	○	○	essential

Comment on subitem 2a-i)

2a-ii) Scientific background, rationale

Scientific background, rationale: What is known about the (type of) system that is the object of the study (be sure to discuss the use of similar systems for other conditions/diagnoses, if appropiate), motivation for the study, i.e. what are the reasons for and what is the context for this specific study, from which stakeholder viewpoint is the study performed, potential impact of findings [2]. Briefly justify the choice of the comparator.

	1	2	3	4	5	
subitem not at all important	○	○	○	○	○	essential

Comment on subitem 2a-ii)

Add a subitem under CONSORT item 2a

2b) Specific objectives or hypotheses

(note: Contrary to STARE-HI we do not recommend to mention IRB approval in this section - JMIR and other journals typically recommend this as a subheading under “methods”. CONSORT-EHEALTH has a separate item for ethical considerations)

(no EHEALTH-specific subitems under CONSORT item 2b)

Comment below to suggest a subitem

METHODS

3a) Description of trial design (such as parallel, factorial) including allocation ratio

(no EHEALTH-specific subitems under CONSORT item 3a)
Comment below to suggest a subitem

3b) Important changes to methods after trial commencement (such as eligibility criteria), with reasons

3b-i) Bug fixes, Downtimes, Content Changes
Bug fixes, Downtimes, Content Changes: ehealth systems are often dynamic systems. A description of changes to methods therefore also includes important changes made on the intervention or comparator during the trial (e.g., major bug fixes or changes in the functionality or content) (5-iii) and other “unexpected events” that may have influenced study design such as staff changes, system failures/downtimes, etc. [2].

	1	2	3	4	5	
subitem not at all important	○	○	○	○	○	essential

Comment on subitem 3b-i)

Add a subitem under CONSORT item 3b

4a) Eligibility criteria for participants

4a-i) Computer / Internet literacy

Computer / Internet literacy is often an implicit “de facto” eligibility criterion - this should be explicitly clarified [1].

	1	2	3	4	5	
subitem not at all important	○	○	○	○	○	essential

Comment on subitem 4a-i)

4a-ii) Open vs. closed, web-based vs. face-to-face assessments:

Open vs. closed, web-based vs. face-to-face assessments: Mention how participants were recruited (online vs. offline), e.g., from an open access website or from a clinic, and clarify if this was a purely web-based trial, or there were face-to-face components (as part of the intervention or for assessment), i.e., to what degree got the study team to know the participant. In online-only trials, clarify if participants were quasi-anonymous and whether having multiple identities was possible or whether technical or logistical measures (e.g., cookies, email confirmation, phone calls) were used to detect/prevent these.

	1	2	3	4	5	
subitem not at all important	○	○	○	○	○	essential

Comment on subitem 4a-ii)

4a-iii) Information giving durnig recruitment

Information given during recruitment. Specify how participants were briefed for recruitment and in the informed consent procedures (e.g., publish the informed consent documentation as appendix, see also item X26), as this information may have an effect on user self-selection, user expectation and may also bias results.

	1	2	3	4	5	
subitem not at all important	○	○	○	○	○	essential

Comment on subitem 4a-iii)

Add a subitem under CONSORT item 4a

4b) Settings and locations where the data were collected

4b-i) Report if outcomes were (self-)assessed through online questionnaires

Clearly report if outcomes were (self-)assessed through online questionnaires (as common in web-based trials) or otherwise.

	1	2	3	4	5	
subitem not at all important	○	○	○	○	○	essential

Comment on subitem 4b-i)

4b-ii) Report how institutional affiliations are displayed

"Report how institutional affiliations are displayed to potential participants [on ehealth media], as affiliations with prestigious hospitals or universities may affect volunteer rates, use, and reactions with regards to an intervention" [1].

	1	2	3	4	5	
subitem not at all important	○	○	○	○	○	essential

Comment on subitem 4b-ii)

Add a subitem under CONSORT item 4b

5) The interventions for each group with sufficient details to allow replication, including how and when they were actually administered

5-i) Mention names, credential, affiliations of the developers, sponsors, and owners
Mention names, credential, affiliations of the developers, sponsors, and owners [6] (if authors/evaluators are owners or developer of the software, this needs to be declared in a "Conflict of interest" section).

	1	2	3	4	5	
subitem not at all important	○	○	○	○	○	essential

Comment on subitem 5-i)

5-ii) Describe the history/development process
Describe the history/development process of the application and previous formative evaluations (e.g., focus groups, usability testing), as these will have an impact on adoption/use rates and help with interpreting results.

	1	2	3	4	5	
subitem not at all important	○	○	○	○	○	essential

Comment on subitem 5-ii)

5-iii) Revisions and updating
Revisions and updating. Clearly mention the date and/or version number of the application/intervention (and comparator, if applicable) evaluated, or describe whether the intervention underwent major changes during the evaluation process, or whether the development and/or content was "frozen" during the trial. Describe dynamic components such as news feeds or changing content which may have an impact on the replicability of the intervention (for

unexpected events see item 3b).

	1	2	3	4	5	
subitem not at all important	○	○	○	○	○	essential

Comment on subitem 5-iii)

5-iv) Quality assurance methods
Provide information on quality assurance methods to ensure accuracy and quality of information provided [1], if applicable.

	1	2	3	4	5	
subitem not at all important	○	○	○	○	○	essential

Comment on subitem 5-iv)

5-v) Ensure replicability by publishing the source code, and/or providing screenshots/screen-capture video, and/or providing flowcharts of the algorithms used
Ensure replicability by publishing the source code, and/or providing screenshots/screen-capture video, and/or providing flowcharts of the algorithms used. Replicability (i.e., other researchers should in principle be able to replicate the study) is a hallmark of scientific reporting.

	1	2	3	4	5	
subitem not at all important	○	○	○	○	○	essential

Comment on subitem 5-v)

5-vi) Digital preservation

Digital preservation: Provide the URL of the application, but as the intervention is likely to change or disappear over the course of the years; also make sure the intervention is archived (Internet Archive, webcitation.org, and/or publishing the source code or screenshots/videos alongside the article). As pages behind login screens cannot be archived, consider creating demo pages which are accessible without login.

	1	2	3	4	5	
subitem not at all important	○	○	○	○	○	essential

Comment on subitem 5-vi)

5-vii) Access

Access: Describe how participants accessed the application, in what setting/context, if they had to pay (or were paid) or not, whether they had to be a member of specific group. If known, describe how participants obtained "access to the platform and Internet" [1]. To ensure access for editors/reviewers/readers, consider to provide a "backdoor" login account or demo mode for reviewers/readers to explore the application (also important for archiving purposes, see vi).

	1	2	3	4	5	
subitem not at all important	○	○	○	○	○	essential

Comment on subitem 5-vii)

5-viii) Mode of delivery, features/functionalities/components of the intervention and comparator, and the theoretical framework

Describe mode of delivery, features/functionalities/components of the intervention and comparator, and the theoretical framework [6] used to design them (instructional strategy [1], behaviour change techniques, persuasive features, etc., see e.g., [7, 8] for terminology). This includes an in-depth description of the content (including where it is coming from and who developed it) [1]," whether [and how] it is tailored to individual circumstances and allows users to track their progress and receive feedback" [6]. This also includes a description of communication delivery channels and – if computer-mediated communication is a component – whether communication was synchronous or asynchronous [6]. It also includes information on presentation strategies [1], including page design principles, average amount of text on pages, presence of hyperlinks to other resources, etc. [1].

	1	2	3	4	5	
subitem not at all important	○	○	○	○	○	essential

Comment on subitem 5-viii)

5-ix) Describe use parameters

Describe use parameters (e.g., intended "doses" and optimal timing for use) [1]. Clarify what instructions or recommendations were given to the user, e.g., regarding timing, frequency, heaviness of use [1], if any, or was the intervention used ad libitum.

	1	2	3	4	5	
subitem not at all important	○	○	○	○	○	essential

Comment on subitem 5-ix)

5-x) Clarify the level of human involvement

Clarify the level of human involvement (care providers or health professionals, also technical assistance) in the e-intervention or as co-intervention (detail number and expertise of professionals involved, if any, as well as "type of assistance offered, the timing and frequency of the support, how it is initiated, and the medium by which the assistance is delivered" [6]. It may be necessary to distinguish between the level of human involvement required for the trial, and the level of human involvement required for a routine application outside of a RCT setting (discuss under item 21 – generalizability).

	1	2	3	4	5	
subitem not at all important	○	○	○	○	○	essential

Comment on subitem 5-x)

5-xi) Report any prompts/reminders used

Report any prompts/reminders used: Clarify if there were prompts (letters, emails, phone calls, SMS) to use the application, what triggered them, frequency etc [1]. It may be necessary to distinguish between the level of prompts/reminders required for the trial, and the level of prompts/reminders for a routine application outside of a RCT setting (discuss under item 21 – generalizability).

	1	2	3	4	5	
subitem not at all important	○	○	○	○	○	essential

Comment on subitem 5-xi)

5-xii) Describe any co-interventions (incl. training/support)

Describe any co-interventions (incl. training/support): Clearly state any “interventions that are provided in addition to the targeted eHealth intervention” [1], as ehealth intervention may not be designed as stand-alone intervention. This includes training sessions and support [1]. It may be necessary to distinguish between the level of training required for the trial, and the level of training for a routine application outside of a RCT setting (discuss under item 21 – generalizability.

	1	2	3	4	5	
subitem not at all important	○	○	○	○	○	essential

Comment on subitem 5-xii)

Add a subitem under CONSORT item 5

6a) Completely defined pre-specified primary and secondary outcome measures, including how and when they were assessed

6a-i) Online questionnaires: describe if they were validated for online use [6] and apply CHERRIES items to describe how the questionnaires were designed/deployed

If outcomes were obtained through online questionnaires, describe if they were validated for online use [6] and apply CHERRIES items to describe how the questionnaires were designed/deployed [9].

	1	2	3	4	5	
subitem not at all important	○	○	○	○	○	essential

Comment on subitem 6a-i)

6a-ii) Describe whether and how "use" (including intensity of use/dosage) was defined/measured/monitored

Describe whether and how "use" (including intensity of use/dosage) was defined/measured/monitored (logins, logfile analysis, etc.). Use/adoption metrics are important process outcomes that should be reported in any ehealth trial.

	1	2	3	4	5	
subitem not at all important	○	○	○	○	○	essential

Comment on subitem 6a-ii)

6a-iii) Describe whether, how, and when qualitative feedback from participants was obtained

Describe whether, how, and when qualitative feedback from participants was obtained (e.g., through emails, feedback forms, interviews, focus groups).

	1	2	3	4	5	
subitem not at all important	○	○	○	○	○	essential

Comment on subitem 6a-iii)

Add a subitem under CONSORT item 6a

6b) Any changes to trial outcomes after the trial commenced, with reasons

(no EHEALTH-specific subitems under CONSORT item 6b)
Comment below to suggest a subitem

7a) How sample size was determined

NPT: When applicable, details of whether and how the clustering by care provides or centers was addressed

7a-i) Describe whether and how expected attrition was taken into account when calculating

the sample size
Describe whether and how expected attrition was taken into account when calculating the sample size.

	1	2	3	4	5	
subitem not at all important	○	○	○	○	○	essential

Comment on subitem 7a-i)

Add a subitem under CONSORT item 7a

7b) When applicable, explanation of any interim analyses and stopping guidelines

(no EHEALTH-specific subitems under CONSORT item 7b)
Comment below to suggest a subitem

8a) Method used to generate the random allocation sequence

NPT: When applicable, how care providers were allocated to each trial group

(no EHEALTH-specific subitems under CONSORT item 8a)
Comment below to suggest a subitem

8b) Type of randomisation; details of any restriction (such as blocking and block size)

(no EHEALTH-specific subitems under CONSORT item 8b)
Comment below to suggest a subitem

9) Mechanism used to implement the random allocation sequence (such as sequentially numbered containers), describing any steps taken to conceal the sequence until interventions were assigned

(no EHEALTH-specific subitems under CONSORT item 9)
Comment below to suggest a subitem

10) Who generated the random allocation sequence, who enrolled participants, and who assigned participants to interventions

(no EHEALTH-specific subitems under CONSORT item 10)
Comment below to suggest a subitem

11a) If done, who was blinded after assignment to interventions (for example, participants, care providers, those assessing outcomes) and how

NPT: Whether or not administering co-interventions were blinded to group assignment

11a-i) Specify who was blinded, and who wasn't
Specify who was blinded, and who wasn't. Usually, in web-based trials it is not possible to blind the participants [1, 3] (this should be clearly acknowledged), but it may be possible to blind outcome assessors, those doing data analysis or those administering co-interventions (if any).

	1	2	3	4	5	
subitem not at all important	○	○	○	○	○	essential

Comment on subitem 11a-i)

11a-ii) Discuss e.g., whether participants knew which intervention was the "intervention of interest" and which one was the "comparator"

Informed consent procedures (4a-ii) can create biases and certain expectations - discuss e.g., whether participants knew which intervention was the "intervention of interest" and which one was the "comparator".

	1	2	3	4	5	
subitem not at all important	○	○	○	○	○	essential

Comment on subitem 11a-ii)

Add a subitem under CONSORT item 11a

11b) If relevant, description of the similarity of interventions

(this item is usually not relevant for ehealth trials as it refers to similarity of a placebo or sham intervention to a active medication/intervention)

(no EHEALTH-specific subitems under CONSORT item 11b)

Comment below to suggest a subitem

12a) Statistical methods used to compare groups for primary and secondary outcomes

NPT: When applicable, details of whether and how the clustering by care providers or centers was addressed

12a-i) Imputation techniques to deal with attrition / missing values
Imputation techniques to deal with attrition / missing values: Not all participants will use the intervention/comparator as intended and attrition is typically high in ehealth trials. Specify how participants who did not use the application or dropped out from the trial were treated in the statistical analysis (a complete case analysis is strongly discouraged, and simple imputation techniques such as LOCF may also be problematic [4]).

	1	2	3	4	5	
subitem not at all important	○	○	○	○	○	essential

Comment on subitem 12a-i)

Add a subitem under CONSORT item 12a

12b) Methods for additional analyses, such as subgroup analyses and adjusted analyses

(no EHEALTH-specific subitems under CONSORT item 12b)

Comment below to suggest a subitem

X26) (not a CONSORT item)

X26-i) Comment on ethics committee approval

	1	2	3	4	5	
subitem not at all important	○	○	○	○	○	essential

Comment on subitem X26-i)

x26-ii) Outline informed consent procedures

Outline informed consent procedures e.g., if consent was obtained offline or online (how? Checkbox, etc.?), and what information was provided (see 4a-ii). See [6] for some items to be included in informed consent documents.

	1	2	3	4	5	
subitem not at all important	◯	◯	◯	◯	◯	essential

Comment on subitem X26-ii)

X26-iii) Safety and security procedures

Safety and security procedures, incl. privacy considerations, and "any steps taken to reduce the likelihood or detection of harm (e.g., education and training, availability of a hotline)" [1].

	1	2	3	4	5	
subitem not at all important	◯	◯	◯	◯	◯	essential

Comment on subitem X26-iii)

Add a subitem under item X26

RESULTS

13a) For each group, the numbers of participants who were randomly assigned, received intended treatment, and were analysed for the primary outcome

NPT: The number of care providers or centers performing the intervention in each group and the number of patients treated by each care provider in each center

(no EHEALTH-specific subitems under CONSORT item 13a)
Comment below to suggest a subitem

13b) For each group, losses and exclusions after randomisation, together with reasons

13b-i) Attrition diagram
Strongly recommended: An attrition diagram (e.g., proportion of participants still logging in or using the intervention/comparator in each group plotted over time, similar to a survival curve) [5] or other figures or tables demonstrating usage/dose/engagement.

	1	2	3	4	5	
subitem not at all important	○	○	○	○	○	essential

Comment on subitem 13b-i)

Add a subitem under CONSORT item 13b

14a) Dates defining the periods of recruitment and follow-up

14a-i) Indicate if critical “secular events” [1] fell into the study period

Indicate if critical “secular events” [1] fell into the study period, e.g., significant changes in Internet resources available or “changes in computer hardware or Interent delivery resources” [1].

	1	2	3	4	5	
subitem not at all important	◯	◯	◯	◯	◯	essential

Comment on subitem 14a-i)

Add a subitem under CONSORT item 14a

14b) Why the trial ended or was stopped (early)

(no EHEALTH-specific subitems under CONSORT item 14b)
Comment below to suggest a subitem

15) A table showing baseline demographic and clinical characteristics for each group

NPT: When applicable, a description of care providers (case volume, qualification, expertise, etc.) and centers (volume) in each group

15-i) Report demographics associated with digital divide issues
In ehealth trials it is particularly important to report demographics associated with digital divide issues, such as age, education, gender, social-economic status, computer/Internet/ehealth literacy of the participants, if known.

	1	2	3	4	5	
subitem not at all important	○	○	○	○	○	essential

Comment on subitem 15-i)

Add a subitem under CONSORT item 15

16) For each group, number of participants (denominator) included in each analysis and whether the analysis was by original assigned groups

16-i) Report multiple “denominators” and provide definitions

Report multiple “denominators” and provide definitions: Report N’s (and effect sizes) “across a range of study participation [and use] thresholds” [1], e.g., N exposed, N consented, N used more than x times, N used more than y weeks, N participants “used” the intervention/comparator at specific pre-defined time points of interest (in absolute and relative numbers per group). Always clearly define “use” of the intervention.

	1	2	3	4	5	
subitem not at all important	○	○	○	○	○	essential

Comment on subitem 16-i)

16-ii) Primary analysis should be intent-to-treat
Primary analysis should be intent-to-treat, secondary analyses could include comparing only "users", with the appropriate caveats that this is no longer a randomized sample (see 18-i).

	1	2	3	4	5	
subitem not at all important	○	○	○	○	○	essential

Comment on subitem 16-ii)

Add a subitem under CONSORT item 16

17a) For each primary and secondary outcome, results for each group, and the estimated effect size and its precision (such as 95% confidence interval)

17a-i) Presentation of process outcomes such as metrics of use and intensity of use
In addition to primary/secondary (clinical) outcomes, the presentation of process outcomes such as metrics of use and intensity of use (dose, exposure) and their operational definitions is critical. This does not only refer to metrics of attrition (13-b) (often a binary variable), but also to more continuous exposure metrics such as "average session length". These must be accompanied by a technical description how a metric like a "session" is defined (e.g., timeout after idle time) [1] (report under item 6a).

	1	2	3	4	5	
subitem not at all important	○	○	○	○	○	essential

Comment on subitem 17a-i)

Add a subitem under CONSORT item 17a

17b) For binary outcomes, presentation of both absolute and relative effect sizes is recommended

(no EHEALTH-specific subitems under CONSORT item 17b)
Comment below to suggest a subitem

18) Results of any other analyses performed, including subgroup analyses and adjusted analyses, distinguishing pre-specified from exploratory

18-i) Subgroup analysis of comparing only users
A subgroup analysis of comparing only users is not uncommon in ehealth trials, but if done, it must be stressed that this is a self-selected sample and no longer an unbiased sample from a randomized trial (see 16-iii).

	1	2	3	4	5	
subitem not at all important	○	○	○	○	○	essential

Comment on subitem 18-i)

Add a subitem under CONSORT item 18

19) All important harms or unintended effects in each group

(for specific guidance see CONSORT for harms)

19-i) Include privacy breaches, technical problems
Include privacy breaches, technical problems. This does not only include physical “harm” to participants, but also incidents such as perceived or real privacy breaches [1], technical problems, and other unexpected/unintended incidents. “Unintended effects” also includes unintended positive effects [2].

	1	2	3	4	5	
subitem not at all important	○	○	○	○	○	essential

Comment on subitem 19-i)

19-ii) Include qualitative feedback from participants or observations from staff/researchers
Include qualitative feedback from participants or observations from staff/researchers, if available, on strengths and shortcomings of the application, especially if they point to unintended/unexpected effects or uses. This includes (if available) reasons for why people did or did not use the application as intended by the developers.

	1	2	3	4	5	
subitem not at all important	○	○	○	○	○	essential

Comment on subitem 19-ii)

Add a subitem under CONSORT item 19

DISCUSSION

22) Interpretation consistent with results, balancing benefits and harms, and considering other relevant evidence

NPT: In addition, take into account the choice of the comparator, lack of or partial blinding, and unequal expertise of care providers or centers in each group

22-i) Restate study questions and summarize the answers suggested by the data [2], starting with primary outcomes and process outcomes (use)
Restate study questions and summarize the answers suggested by the data [2], starting with primary outcomes and process outcomes (use).

	1	2	3	4	5	
subitem not at all important	○	○	○	○	○	essential

Comment on subitem 22-i)

22-ii) Highlight unanswered new questions, suggest future research [2]
Highlight unanswered new questions, suggest future research [2].

	1	2	3	4	5	
subitem not at all important	○	○	○	○	○	essential

Comment on subitem 22-ii)

Add a subitem under CONSORT item 22

20) Trial limitations, addressing sources of potential bias, imprecision, and, if relevant, multiplicity of analyses

20-i) Typical limitations in ehealth trials

Typical limitations in ehealth trials: Participants in ehealth trials are rarely blinded. Ehealth trials often look at a multiplicity of outcomes, increasing risk for a Type I error. Discuss biases due to non-use of the intervention/usability issues, biases through informed consent procedures, unexpected events.

	1	2	3	4	5	
subitem not at all important	○	○	○	○	○	essential

Comment on subitem 20-i)

Add a subitem under CONSORT item 20

21) Generalisability (external validity, applicability) of the trial findings

NPT: External validity of the trial findings according to the intervention, comparators, patients, and care providers or centers involved in the trial

21-i) Generalizability to other populations

Generalizability to other populations: In particular, discuss generalizability to a general Internet population, outside of a RCT setting, and general patient population, including applicability of the study results for other organizations [2].

	1	2	3	4	5	
subitem not at all important	○	○	○	○	○	essential

Comment on subitem 21-i)

21-ii) Discuss if there were elements in the RCT that would be different in a routine application setting

Discuss if there were elements in the RCT that would be different in a routine application setting (e.g., prompts/reminders, more human involvement, training sessions or other co-interventions) and what impact the omission of these elements could have on use, adoption, or outcomes if the intervention is applied outside of a RCT setting.

	1	2	3	4	5	
subitem not at all important	○	○	○	○	○	essential

Comment on subitem 21-ii)

Add a subitem under CONSORT item 21

OTHER INFORMATION

23) Registration number and name of trial registry

(no EHEALTH-specific subitems under CONSORT item 23)
Comment below to suggest a subitem

24) Where the full trial protocol can be accessed, if available

(no EHEALTH-specific subitems under CONSORT item 24)
Comment below to suggest a subitem

25) Sources of funding and other support (such as supply of drugs), role of funders

(no EHEALTH-specific subitems under CONSORT item 25)
Comment below to suggest a subitem

X27) (not a CONSORT item)

X27-i) State the “relation of the study team towards the system being evaluated”
In addition to the usual declaration of interests (financial or otherwise), also state the “relation of the study team towards the system being evaluated” [2], i.e., state if the authors/evaluators are distinct from or identical with the developers/sponsors of the intervention.

	1	2	3	4	5	
subitem not at all important	○	○	○	○	○	essential

Comment on subitem X27-i)

Add a subitem under item X27

Last question

Do you want to become involved in the writing committee working on the elaboration document? If yes, please provide the subitems you wish to elaborate on

e.g., 3b-i, 5

Submit

Powered by Google Docs

Report Abuse - Terms of Service - Additional Terms
