# Supplementary material for: CONSORT-EHEALTH: Improving and Standardizing Evaluation Reports of Web-based and Mobile Health Interventions
Source: J Med Internet Res. 2011 Dec 31;13(4):e126. doi: 10.2196/jmir.1923 (PMC3278112; doi:10.2196/jmir.1923)

55 responses

Summary See complete responses

Do you want to be acknowledged (with your name and affilitation) in the CONSORT-EHEALTH publication?

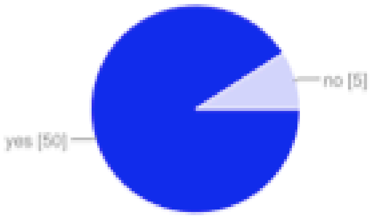

yes	**50**	91%
no	**5**	9%

Your role/experience with ehealth-trials

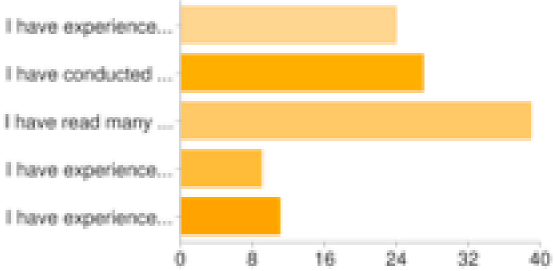

I have experience with conducting ehealth studies myself, but no I
I have conducted ehealth RCTs
I have read many ehealth RCT/evaluation reports
I have experience mainly from a consumer/patient point of view
I have experience mainly from a policy/implementation/decision-m

People may select more than one checkbox, so percentages may

Other guidelines

I have conducted RCT drug trials under CONSORT guidelines. Reported using STROBE guidelines for cohort studies. No, these proposed guidelines that I have seen dealing with eHealth studies. Just the ones mentioned at the IRSII CONSORT workshop I have a suggestion. This approach

TITLE AND ABSTRACT

1a) Identification as a randomized trial in the title

1a-i) Identify the mode of delivery in the title

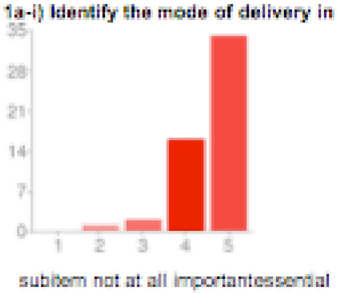

1 -subitem not at all important	**0**	0%
2	**1**	2%
3	**2**	4%
4	**16**	29%
5 -essential	**34**	62%

Comment on subitem 1a-i)

Standardization of terminology is essential. I have favored "Internet-based" over "web-based" because web pages are not necessarily accessed over the Internet, but the latter term seems more precise if we are defining "web-based" as accessed over the World-Wide Web. Perhaps HTML-based "web pages" that are not accessed over the World-Wide Web should simply be called "HTML-based." I would recommend not using Internet-based as a separate term, but rather specify this; e.g., web-based with e-mail support (instead of Internet-based). The inclusion of your clarification of "web-based" "online" etc a ...

1a-ii) Non-web-based components or important co-interventions in title

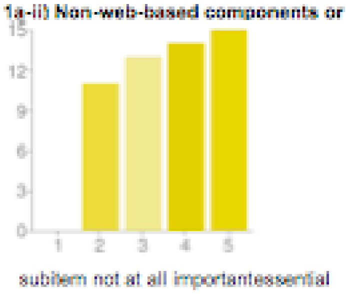

1 -subitem not at all important	**0**	0%
2	**11**	20%
3	**13**	24%
4	**14**	25%
5 -essential	**15**	27%

Comment on subitem 1a-ii)

To the extent that titles are limited in length, I see this as less essential than identifying the primary modality and target group. This will vary with the importance of the non-web based component - if a 24 hour help line number is listed but no one uses it? If the title becomes too long by including the non-web-based components, these should be included in the abstract and described in the manuscript. To allow others who are not e-halth proffesional to understand our results. with tablet PC support Similar comment, essential to remove possible confounding factors Possible to add: Personal

1a-iii) Primary condition or target group in the title

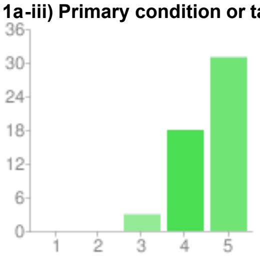

subitem not at all importantessential

1 -subitem not at all important	**0**	0%
2	**0**	0%
3	**3**	5%
4	**18**	33%
5 -essential	**31**	56%

Comment on subitem 1a-iii)

If primary condition/target group is not mentioned in the title, it is absolutely essential to mention in the abstract. I would recommend requiring both primary condition AND the target group. If the title becomes too long with the target group(my RCT was directed to generally healthy adults), this should be clearly described in the abstract and manuscript. I think it is important to phrase in terms of the WHO ICF. An mobile EHR with tablet PC support for patients with mental disabilities Essential to allow for future comparisons across studies and allow the possibility of being part of meta-ana ...

Add a subitem under CONSORT item 1a

Preferably some sort of intervention description. May only apply to E-mental health (e.g. "Randomized trial of web-based CBT for elderly academics with depression") Mention if the intervention is for individuals or groups. Mention if the intervenion is educational (information only) or therapeutical. Unlike the traditional randomized clinical trials and due to psychosocial factors and cultural differences between countries. I believe that it would be important to include the place/country where the interventions is made in title (eg, "in Spain") I suggest using acronyms for the RCTs that use mH ...

1b) Structured summary of trial design, methods, results, and conclusions

NPT extension: Description of experimental treatment, comparator, care providers, centers, and blinding status.

1b-i) Key features/functionalities/components of the intervention and comparator in the abstract

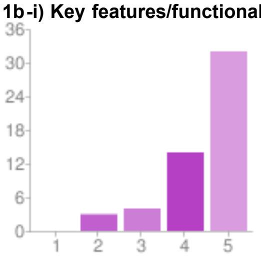

subitem not at all importantessential

1 -subitem not at all important	**0**	0%
2	**3**	5%
3	**4**	7%
4	**14**	25%
5 -essential	**32**	58%

Comment on subitem 1b-i)

Standardizing terminology (as in 1a-i) also seems essential for key features and functionalities. Here, should we also complement or substitute product names with broader terms for the class of products (such as “multimedia” instead of “Flash”)? It is more important to have a more elaborate description of intervention features in the body to help identify

what makes an intervention work/not work. I would recommend requiring the description of theories used. key features/functionalities/components of the intervention and comparator: this is essential theories and principles used for designing ...

1b-ii) Level of human involvement in the abstract

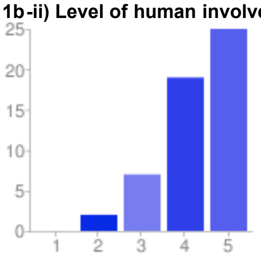

subitem not at all importantessential

1 -subitem not at all important	**0**	0%
2	**2**	4%
3	**7**	13%
4	**19**	35%
5 -essential	**25**	45%

Comment on subitem 1b-ii)

"fully automated" vs. "therapist/nurse/care provider/physician-assisted" : essential mention number and expertise of providers involved: low priority for abstract (score 1) therapist/physician assisted Essential to allow for future comparisons across studies and allow the possibility of being part of meta-analyses Overall level of human involvement = Essential in abstract Number and expertise of providers = Perhaps no space in abstract! The example can be completed in this way: "therapist/nurse/care provider/physician/patient-assisted" I don't think that mentioning number of providers involve ...

1b-iii) Open vs. closed, web-based (self-assessment) vs. face-to-face assessments in abstract

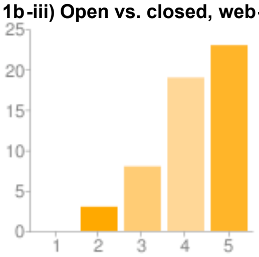

subitem not at all importantessential

1 -subitem not at all important	**0**	0%
2	**3**	5%
3	**8**	15%
4	**19**	35%
5 -essential	**23**	42%

Comment on subitem 1b-iii)

What about mixed designs using both open and closed recruitment methods? I have concerns that this may be too limiting in terms of "self-assessment" eg if all participants were provided with remote telemetry or if the study uses data from GPS is this still 'self-assessment. Can this be clarified here? Open v closed recruitment: essential web-based v face-to-face components: essential Interviewer based assessments are commonly performed via telephone. This is really important. I conducted a review on eHealth applications myself and it was hard sometimes to compare results, because recruitment ...

1b-iv) Results in abstract must contain use data

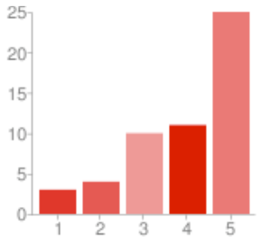

subitem not at all importantessential

1 -subitem not at all important	**3**	5%
2	**4**	7%
3	**10**	18%
4	**11**	20%
5 -essential	**25**	45%

Comment on subitem 1b-iv)

It would be challenging to do an adequate job of describing use metrics in an abstract. Without important details, use metrics can be misleading. Identifying primary and secondary outcomes in the abstract, on the other hand, is essential. participants enrolled/assessed in each group: essential 5 the use/uptake of the intervention (e.g., attrition/adherence metrics, use over time, number of logins etc. - Essential for the key endpoint eg at/for 12 months (score 5) primary/secondary outcomes: essential 5 I would make clear the distinction between adherence to treatment from lost-to-follow-up ...

1b-v) Conclusions/Discussions in abstract for negative trials

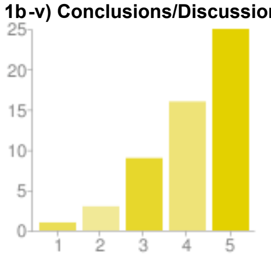

subitem not at all importantessential

1 -subitem not at all important	**1**	2%
2	**3**	5%
3	**9**	16%
4	**16**	29%
5 -essential	**25**	45%

Comment on subitem 1b-v)

Again, it seems like it would be challenging to do an adequate job of "discussing" possible reasons for a negative result in the abstract. Could this be simplified to recommend that authors cite the supposed primary reason for a negative result in the abstract and discuss that and possible alternatives in the discussion? I would recommend to also describe the impact of intervention use if the primary outcome did change. The comment on what to discuss if the trial is negative seems overly specific. There are a multitude of ways that discussions can be misinterpretations. Maybe make more genera ...

Add a subitem under CONSORT item 1b

Primary condition or target group in the abstract (see 1a-iii above) Response rates of assessment / data collection should be disclosed in the abstract (eg. At 3 months, the follow-up response rate was 74%.). Clinical Trial Registration number e.g. http://www.anzctr.org.au In all aspects relating to the abstract I am aware of the very tight word limits in some journals (versus the needs of readers potential systematic reviewers) The minimum system/infrastructure requirements for planning and conducting such an intervention in other contexts should be written in an itemized format; like the i ...

INTRODUCTION

2a) Scientific background and explanation of rationale

2a-i) Problem and the type of system/solution

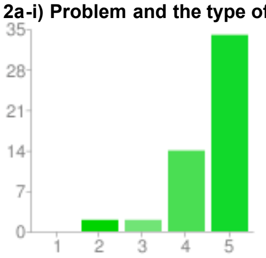

subitem not at all importantessential

1 -subitem not at all important	**0**	0%
2	**2**	4%
3	**2**	4%
4	**14**	25%
5 -essential	**34**	62%

Comment on subitem 2a-i)

In this sense it is very important that is becomes clear that developing a web-based interventions was goal-driven instead of tool-driven. I find often that studies focus too much on the technology, while the goal behind it should be the drive for developing the intervention. mention the traditional interventions for the particular patient population and the advantages of the new system A significant number of people with mental diseases remain unnoticed due to the incorrect identification of the symptomatology, the resistance to seek either help or information regarding these services, among ...

2a-ii) Scientific background, rationale

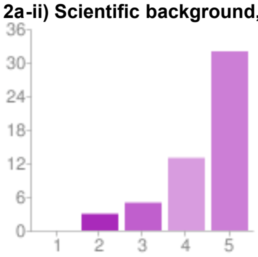

subitem not at all importantessential

1 -subitem not at all important	**0**	0%
2	**3**	5%
3	**5**	9%
4	**13**	24%
5 -essential	**32**	58%

Comment on subitem 2a-ii)

Accessing to EHRs through mobile devices provides a number of advantages both for health centers and clinical staff, and for patients. Among these advantages are: accessing to patients' information in real time (from wherever and whenever), resource savings, improving the information management, and reducing the delay in health care. In the field of mental health, there are important epidemiological studies releasing relevant information about types and rates of more frequent disorders. However, a significant number of people with mental diseases remain unnoticed due to the incorrect identif ...

Add a subitem under CONSORT item 2a

Possibility of wider implementation of the system. No Additions. No. need to clearly state what gap this study addresses, and how it contributes to knowledge

2b) Specific objectives or hypotheses

(note: Contrary to STARE-HI we do not recommend to mention IRB approval in this section - JMIR and other journals typically recommend this as a subheading under “methods”. CONSORT-EHEALTH has a separate item for ethical considerations)

(no EHEALTH-specific subitems under CONSORT item 2b)

I assume there is, however, a requirement for specific objectives. Essential to allow for future comparisons across studies and allow the possibility of being part of meta-analyses No comments. I agree that IRB approval should appear in Methods only No. I agree - ethics belong in the method section ift not a part of the research question(s) I concur that IRB/ethical issues should appear in Methods, not Intro.

METHODS

3a) Description of trial design (such as parallel, factorial) including allocation ratio

(no EHEALTH-specific subitems under CONSORT item 3a)

I agree, this is often too complex to perform in ehealth trials That will be mentioned except for the allocation ratio because the allocation was coded randomized during the programming process (and normally it is not a rule to provide the algorithm) mere mention of the design and allocation ratio would be ideal. No. need to mention whether there's follow-up, and for how long How will this guideline relate to extensions for non-inferiority, cluster, etc.?

3b) Important changes to methods after trial commencement (such as eligibility criteria), with reasons

3b-i) Bug fixes, Downtimes, Content Changes

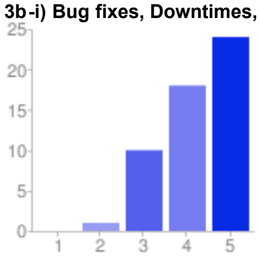

subitem not at all importantessential

1 -subitem not at all important	**0**	0%
2	**1**	2%
3	**10**	18%
4	**18**	33%
5 -essential	**24**	44%

Comment on subitem 3b-i)

Especially the reasons are important to elaborate on. Conventional trials may have 'down time' etc which I would expect to be reported if they were significant, so major breaks should be reported. However, major changes to functionality or content or design would be essetial to report (score 5) Authors should mention if new content was added to system during the trial. In my opinion this is often the case. Essential to

allow for future comparisons across studies and allow the possibility of being part of meta-analyses Such change events can be represented in a timeline fashion. I think that "u ...

Add a subitem under CONSORT item 3b

Brief description of website layout and complexity ("After a brief welcome screen, participants enter their weight and length and proceed to the food diary in the taskbar etc.") More specifics regarding the software used during the interventions and whatever was used for analysis. After completion of trials, there may be a "lag" time for researchers to prepare and publish the results of their study. It may happen that the version(s) of the solutions used at the time of study design and conduct, change during this "lag" time. The discussion on these kinds of "version" changes seems necessary. T ...

4a) Eligibility criteria for participants

4a-i) Computer / Internet literacy

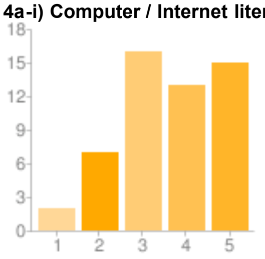

subitem not at all importantessential

1 -subitem not at all important	**2**	4%
2	**7**	13%
3	**16**	29%
4	**13**	24%
5 -essential	**15**	27%

Comment on subitem 4a-i)

Only to be stated and assessed if there is reason to suspect that a target group may be illiterate in the world of technology. For example, studying a group of ADHD patients where dyslexia is a highly prevalent condition or older participants with age >60 years. I consider this part of the needs assessment and this should only be mentioned in the discussion section if this appears to be a problem. Low importance in open trials e.g. users have found the site. However, in closed trials this may be an important factor e.g. if they are referred to the site for treatment. In both types of trial, t ...

4a-ii) Open vs. closed, web-based vs. face-to-face assessments:

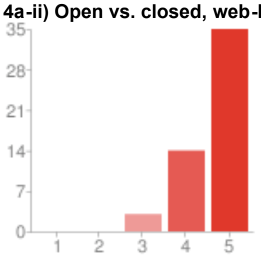

subitem not at all importantessential

1 -subitem not at all important	**0**	0%
2	**0**	0%
3	**3**	5%
4	**14**	25%
5 -essential	**35**	64%

Comment on subitem 4a-ii)

An important point is that it may be extremely difficult or even impossible for researchers to have a complete overview of the entire recruitment process due to the fact that, at least, the Internet is a medium which is hard to control. One such

example which serves to identify many of the problems I mention is viral recruitment where a researcher uses his or others' social networks to recruit participants. The researcher can control his 1st degree contacts, but this is hardly possible for 2nd, 3rd... degree contacts. That is simply the nature of online viral recruitment. This is similar to ...

4a-iii) Information giving durnig recruitment

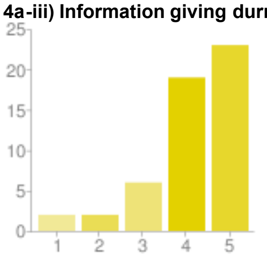

subitem not at all importantessential

1 -subitem not at all important	**2**	4%
2	**2**	4%
3	**6**	11%
4	**19**	35%
5 -essential	**23**	42%

Comment on subitem 4a-iii)

I see publishing informed consenst as appendix as a bit redundant, because many informed consents will have to be translated to English which obviously is problematic and in a way biased because of challenges with translation. However, I would highly appreciate that researchers state in what sequence information was given, briefly state what information was given, how many contact points there were with researcher, etc. While this information may be useful/interesting, until all journals allow on-line supplements it can not be an essential requirement. Yes! I think this information should be ...

Add a subitem under CONSORT item 4a

If available, clarify which/why eligible participants declined to enter the trial (demographics, etc.) As many definitions or synonyms appear in literature for ehealth interventions (such as web-based, health technology, health information technology, internet-based, ehealth etc), it must be made clear what definition the authors use and why. This makes it easier in reviews to compare studies to one another. Nothing specific remains. No. Would be good to highline strategy for adherence I suggest extending the flowchart to begin with 'participants approached' so the recruitment process is clear.

4b) Settings and locations where the data were collected

4b-i) Report if outcomes were (self-)assessed through online questionnaires

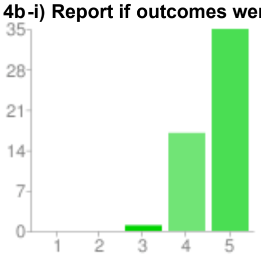

subitem not at all importantessential

1 -subitem not at all important	**0**	0%
2	**0**	0%
3	**1**	2%
4	**17**	31%
5 -essential	**35**	64%

Comment on subitem 4b-i)

Data collection instruments and methods should be described I would ask for clarification on 1) the assessment medium (online, mail, telephone interview, etc.) and 2) perspective (self-report, interviewer, etc.) If possible, comment on how successful the adaptation of an existing pen-and-paper to an online questionnaire was (e.g., visual analogue scale required with pen-and-paper test but was not available in online questionnaire software, etc.) Essential to allow for future comparisons across studies and allow the possibility of being part of meta-analyses Essential Details of pilot-testing of ...

4b-ii) Report how institutional affiliations are displayed

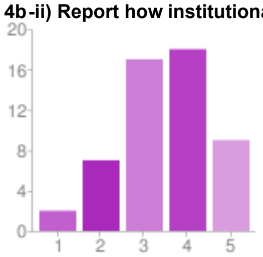

subitem not at all importantessential

1 -subitem not at all important	**2**	4%
2	**7**	13%
3	**17**	31%
4	**18**	33%
5 -essential	**9**	16%

Comment on subitem 4b-ii)

This is common to face-to-face interventions where universities / hospitals may be regarded as more trust worthy than drug companies. This is the issue of the credibility of website or mobile intervention. It is not unique to ehealth (a pharmacotherapy trial by Harvard may be perceived differently than one run out of a private practice). This seems to be adding a new criterion category. Essential to allow for future comparisons across studies and allow the possibility of being part of meta-analyses A very nice idea This is an interesting aspect, however, I don't think it's an essential item. W ...

Add a subitem under CONSORT item 4b

If not self-assessed, it is needed to describe (a) how participants were assessed and (b) whether this was problematic given the setting of a web-based intervention. Are online questionnaire metrics reported, and how were "unlikely" questionnaire metrics handled (e.g., were questionnaires that were answered unusually quickly included (indicative of participant inattention or laziness)) I am not sure if this is the right heading for this item. However, to my opinion it is important to state how participants got to know about the intervention. How was it spread/promoted to potential users? Often ...

5) The interventions for each group with sufficient details to allow replication, including how and when they were actually administered

5-i) Mention names, credential, affiliations of the developers, sponsors, and owners

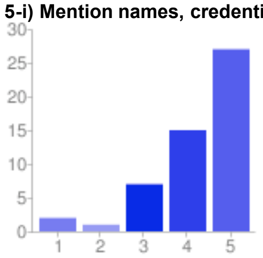

subitem not at all importantessential

1 -subitem not at all important	**2**	4%
2	**1**	2%
3	**7**	13%
4	**15**	27%
5 -essential	**27**	49%

Comment on subitem 5-i)

CoI section essential (score 5) but this bias exists in all intervention types. I'm not clear how this differs from the conflict of interest issue. This should be done through contact with the first or corresponding author, not through the manuscript. Essential to allow for future comparisons across studies and allow the possibility of being part of meta-analyses Sponorship should always be clearly disclosed as it can impact results and intepretation of findings Essential I think the cost(s) of developing (and/or acquiring) the software (especially the direct costs) and also software availabili ...

5-ii) Describe the history/development process

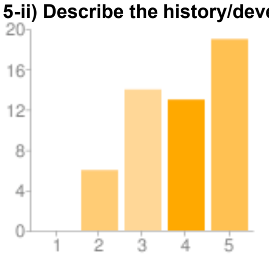

subitem not at all importantessential

1 -subitem not at all important	**0**	0%
2	**6**	11%
3	**14**	25%
4	**13**	24%
5 -essential	**19**	35%

Comment on subitem 5-ii)

Essential unless these were published previously elsewhere. Including a logical/process model of the intervention, for example in a flow chart, would really enhance the publications. It may be necessary to add this in an appendix. There should preferrably be some guidelines on how to develop a logical/process model. This is very important to for designers, practitioners, and academics to learn more about the success/pitfalls of intervention design and development. Also describe how formative evaluations shaped the intervention. If these are not previously published, I'm not sure that saying " ...

5-iii) Revisions and updating

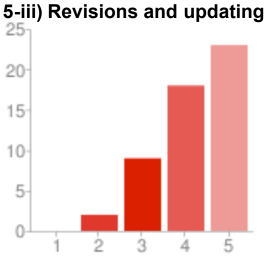

subitem not at all importantessential

1 -subitem not at all important	**0**	0%
2	**2**	4%
3	**9**	16%
4	**18**	33%
5 -essential	**23**	42%

Comment on subitem 5-iii)

This may be difficult, especially for "social" networking-based interventions with a lot of user generated content. Such interventions will change continuously as a direct consequence of the use of these interventions by the participants - something uncontrolled by the researcher / project leader, and probably even difficult to track / disclose in a publication. this is absolutely important when talking about internet interventions. They are dynamic. It should be stated clearly how components could have changed. Essential to allow for future comparisons across studies and allow the possibilit ...

5-iv) Quality assurance methods

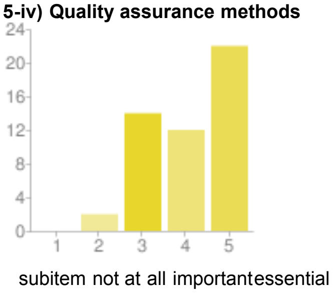

1 -subitem not at all important	**0**	0%
2	**2**	4%
3	**14**	25%
4	**12**	22%
5 -essential	**22**	40%

Comment on subitem 5-iv)

There should be examples of what counts as a quality assurance method. Need more information on this point to assess its importance. Do you mean whether the information was created by health personnel, verified by experts, etc.? I'm not clear what is being asked here. Is it the quality of the static content? or the quality of feedback provided (e.g. when an intervention performs assessments and feedback as part of the intervention, if validated assessment tools are used)? Or implementation? Quality assurance is up to the PI, who should be following ethical standards. I can't imagine how this ...

5-v) Ensure replicability by publishing the source code, and/or providing screenshots/screen-capture video, and/or providing flowcharts of the algorithms used

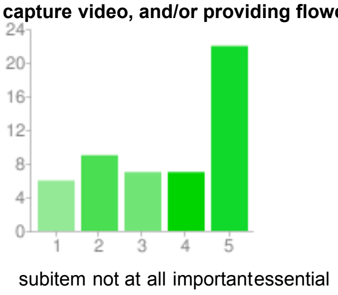

1 -subitem not at all important	**6**	11%
2	**9**	16%
3	**7**	13%
4	**7**	13%
5 -essential	**22**	40%

Comment on subitem 5-v)

I am a psychologist and Research Scientist for a small business. Publishing our source code is not something the owners would allow. Replicability should hinge on the theory guiding the development of the intervention and the hypothesized active ingredients. It should be up to the replicators to develop something comparable, sufficient detail should be provided to enable this, but not the original source code. Source code should be proprietary. In the name of science and research, I completely understand why we would want researchers to publish source code and algorithms. If it does not p ...

5-vi) Digital preservation

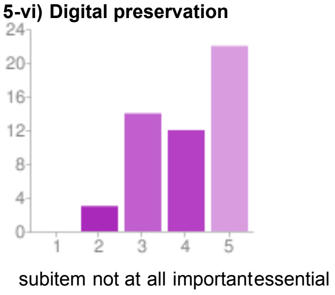

1 -subitem not at all important	**0**	0%
2	**3**	5%
3	**14**	25%
4	**12**	22%
5 -essential	**22**	40%

Comment on subitem 5-vi)

As with publishing the source code, it is not always possible to digitally archive an intervention. Again, sufficient detail should be provided to enable readers to understand all the components of the intervention to replicate or build upon prior work. If this is a funded research project, the URL will be inactive by the time the manuscript is published. This digital preservation is not likely or even possible in many cases. It would be a "demo" only - which is not very helpful. see my comment above. Essential to allow for future comparisons across studies and allow the possibility of being ...

5-vii) Access

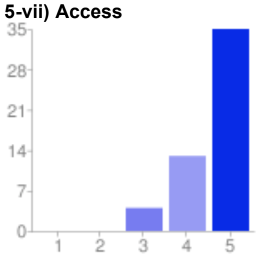

subitem not at all importantessential

1 -subitem not at all important	**0**	0%
2	**0**	0%
3	**4**	7%
4	**13**	24%
5 -essential	**35**	64%

Comment on subitem 5-vii)

"backdoor" login account or demo mode for reviewers/readers to explore the application (also important for archiving purposes..)" This would be absolutely wonderful. Participant access needs to be described - essentail (score 5) This item seems to confound participant access and reviewer access. I think separating out the requirements of reporting about the intervention from the methods of disclosure would clarify. Essential to allow for future comparisons across studies and allow the possibility of being part of meta-analyses Essential Any specific constraint in access should be discussed; fo ...

5-viii) Mode of delivery, features/functionalities/components of the intervention and comparator, and the theoretical framework

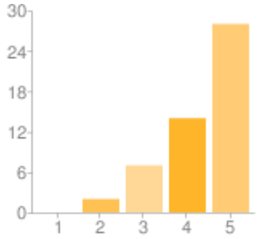

subitem not at all importantessential

1 -subitem not at all important	**0**	0%
2	**2**	4%
3	**7**	13%
4	**14**	25%
5 -essential	**28**	51%

Comment on subitem 5-viii)

See my comments on 5-ii) Describe the history/development process Yes! I would also include 1) descriptions of interactive tools (data input from users, how data were manipulated, and output to patient). 2) automated monitoring/feedback (visual, quantified or qualitative feedback). Trying to grasp the workings of a website with the minimum of information provided by most articles is almost impossible. Vital!!! Elements of this section as appropriate to the particular study. A taxonomy for the different 'ingredients' of the interventions would be useful. However, ehealth interventions often hav ...

5-ix) Describe use parameters

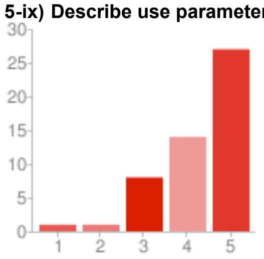

subitem not at all importantessential

1 -subitem not at all important	**1**	2%
2	**1**	2%
3	**8**	15%
4	**14**	25%
5 -essential	**27**	49%

Comment on subitem 5-ix)

Yes! If there is paradata available, also report the extent to which intended doses compared with actual use. exposure is very important!! Essential to allow for future comparisons across studies and allow the possibility of being part of meta-analyses Essential I think a few measures of COMPLIANCE to the intervention should be mentioned in this part as an example. I imagine this would be reported as needed, and doesn't need to be a guideline. In the intervention section only, can be showcased with a pictorial representation or a flow diagram. idem This is an important point but may very likely to b ...

5-x) Clarify the level of human involvement

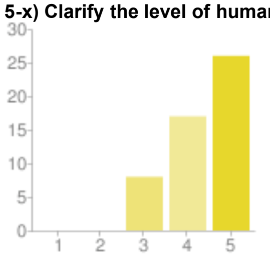

subitem not at all importantessential

1 -subitem not at all important	**0**	0%
2	**0**	0%
3	**8**	15%
4	**17**	31%
5 -essential	**26**	47%

Comment on subitem 5-x)

Also, identify any manuals, procedures or principals used in guiding the human support. this might especially provide important information for implementation strategies of ehealth interventions. It might be that in the beginning more human involvement is needed. Essential to allow for future comparisons across studies and allow the possibility of being part of meta-analyses Essential High-risk alerts of participants that may harm themselves etc. and how these were dealt with (automated, human-intervention etc). In the intervention section. idem Clear explanation is required, as this item can be s ...

5-xi) Report any prompts/reminders used

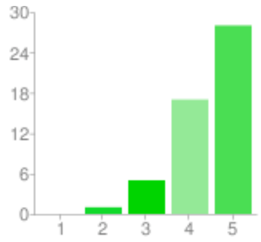

subitem not at all importantessential

1 -subitem not at all important	**0**	0%
2	**1**	2%
3	**5**	9%
4	**17**	31%
5 -essential	**28**	51%

Comment on subitem 5-xi)

I would recommend requiring to describe in general (not only prompts/reminders) which methods/strategies are used to facilitate use: first visit, staying and revisiting. Also, report what the prompts/reminders were meant to accomplish The content of the prompts is also essential. Prompts may be extremely reinforcing the use of the intervention. Essential to allow for future comparisons across studies and allow the possibility of being part of meta-analyses Very important These ways of reminding have different effects in different contexts and for different interventions. How one can assure the re ...

5-xii) Describe any co-interventions (incl. training/support)

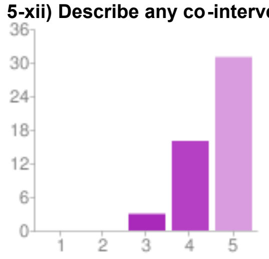

subitem not at all importantessential

1 -subitem not at all important	**0**	0%
2	**0**	0%
3	**3**	5%
4	**16**	29%
5 -essential	**31**	56%

Comment on subitem 5-xii)

It sounds like you mean training/support to the patient, not a coach. I'd just clarify in the title. Essential to allow for future comparisons across studies and allow the possibility of being part of meta-analyses Very important Number and duration of the training sessions are important issues to consider. The trainers' skills should be part of the discussion in this part. A few challenges still remain here: if an intervention is defined as using a "drug", we have biochemical tests to assess its therapeutic dosage in the participants; is there any such test(s) that can tell us a training has ...

Add a subitem under CONSORT item 5

See my comments on 5-ii) Describe the history/development process on adding a subitem on logical/process models. Many interventions use progression rules. Some information on how people move through a staged intervention (e.g. are they allowed to move one only upon completion of a set of material [activity based rules], or after a certain time period [time based rules], or based on acheivement of their own goals, or upon a coach/therapist determination, or was the entire intervention open? This could be embedded above rather than creating a new item Also provide details about how the co-inter ...

6a) Completely defined pre-specified primary and secondary outcome measures, including how and when they were assessed

6a-i) Online questionnaires: describe if they were validated for online use [6] and apply CHERRIES items to describe how the questionnaires were designed/deployed

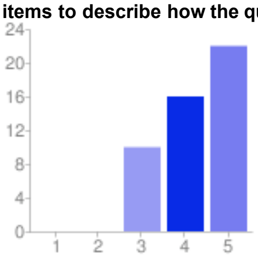

subitem not at all importantessential

1 -subitem not at all important	**0**	0%
2	**0**	0%
3	**10**	18%
4	**16**	29%
5 -essential	**22**	40%

Comment on subitem 6a-i)

Although important, it should not be a panacea whether a measure or scale has been validated for online use, as many measures and scales have not. CHERRIES contains some 30 items, and although it is a truly valuable checklist for online surveys, the inclusion of 30 more items in the current, already extensive CONSORT-EHEALTH checklist may become conflicting with the maximum length of research papers for a large proportion of Journals. Maybe a selection of the most relevant CHERRIES items is possible? I do not know what CHERRIES items are. You should have defined/explained the term! Essential t ...

6a-ii) Describe whether and how "use" (including intensity of use/dosage) was defined/measured/monitored

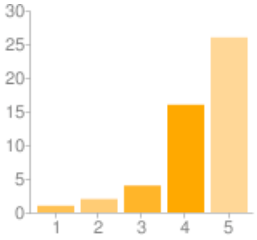

subitem not at all importantessential

1 -subitem not at all important	**1**	2%
2	**2**	4%
3	**4**	7%
4	**16**	29%
5 -essential	**26**	47%

Comment on subitem 6a-ii)

Although it is considered important and should be published, it should not be applied uncritically. A large body of literature in information systems research (see for example Straub et al. (1993) "measuring system usage") which shows how problematic measures of usage actually can be. This is a critical item. My sense is a lot of this gets buried. For example, people often use "module completion" as a criterion. But that is not defined. In some cases it just means they downloaded a pdf, but it is not clear that the module was read. That is different from seeing that someone went through al ...

6a-iii) Describe whether, how, and when qualitative feedback from participants was obtained

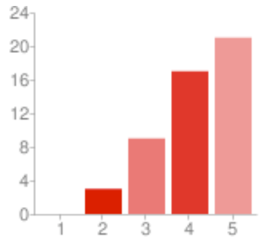

1 -subitem not at all important	**0**	0%
2	**3**	5%
3	**9**	16%
4	**17**	31%
5 -essential	**21**	38%

Comment on subitem 6a-iii)

Only if appropriate for the study. Essential to allow for future comparisons across studies and allow the possibility of being part of meta-analyses Important if analyzed - Statistical summary It has to be described and the results of the qualitative analyses can be tabulated to show the trend. Good point. This is unclear and, I think, not essential. I'm not sure if this means information about acceptability of the trial or the development of the site? In either case, I think qualitative research should be reported following appropriate methods and standards for reporting. This doesn't seem to ...

Add a subitem under CONSORT item 6a

It might be that also some unexpected side effects could be detected. researchers should acknowledge the value of this for the young ehealth research field. These should also be stated when encountered. - No.

6b) Any changes to trial outcomes after the trial commenced, with reasons

(no EHEALTH-specific subitems under CONSORT item 6b)

It might be that also some unexpected side effects could be detected. researchers should acknowledge the value of this for the young ehealth research field. These should also be stated when encountered. It could provide important clues for future trials what we could expect to be outcomes of an intervention. Will be obviously mentioned. No. esp. if participants reported adverse events Agreed

7a) How sample size was determined

NPT: When applicable, details of whether and how the clustering by care provides or centers was addressed

7a-i) Describe whether and how expected attrition was taken into account when calculating the sample size

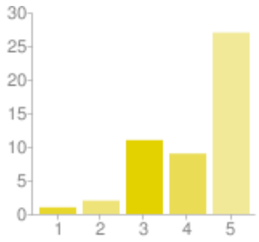

subitem not at all importantessential

1 -subitem not at all important	**1**	2%
2	**2**	4%
3	**11**	20%
4	**9**	16%
5 -essential	**27**	49%

Comment on subitem 7a-i)

This seems primarily important in the context of a negative result. If a significant effect was not found, was it because there was not sufficient power to detect an effect. If a significant effect was found, it seems like a moot point. This is somewhat dependent on the statistical analysis method used. Many account for loss-to-follow-up. Also, cite relevant sources of previous trials for a well-founded estimate of attrition It's important, perhaps essential, that power analysis be conducted by the investigator when the trial is designed, but I don't believe the description of how that was don ...

Add a subitem under CONSORT item 7a

Sampling method (cluster, stratified, etc.) and sampling frame should be discussed. - No.

7b) When applicable, explanation of any interim analyses and stopping guidelines

(no EHEALTH-specific subitems under CONSORT item 7b)

(Note at ISRII several people reported interim analyses - none memtion if this was pre-specied in the protocol or what statistical adjustemnt was made for these 'additional' analayses). Yes, something should be included about this. Maybe this should be included as part of the methodology since interim analyses would be useful and probably hold more weight than, say, post-hoc analyses. Stopping guidelines would be useful as well if researchers can anticipate what negative outcomes might occur as a result of conducting the study - No. Agreed

8a) Method used to generate the random allocation sequence

NPT: When applicable, how care providers were allocated to each trial group

(no EHEALTH-specific subitems under CONSORT item 8a)

Yes, this should be included. Any method that was used to determine how participants were recruited would be essential Yes - Randomization approach should be described. Discussed already in the methodology section. within the trial of some e-health applications randomization and allocation can be done at the same time by computer just at the moment of use of the application (exp.or control) without pre generating an allocation sequence. This should be mentioned specifically because allocation concealement is of course fully respected in that case. No. Agreed

8b) Type of randomisation; details of any restriction (such as blocking and block size)

(no EHEALTH-specific subitems under CONSORT item 8b)

Yes, this should be included. A good methodology would allow for evaluation throughout   if applicable, should be included in the methodology section only.   No.   Agreed, though the relationship between this and other guidelines should be clarified.

9) Mechanism used to implement the random allocation sequence (such as sequentially numbered containers), describing any steps taken to conceal the sequence until interventions were assigned

(no EHEALTH-specific subitems under CONSORT item 9)

Yes, this should be included   Should be mentioned in the Methodology subsection.   see above 8a   No.   Agreed, though perhaps use examples appropriate to e-health

10) Who generated the random allocation sequence, who enrolled participants, and who assigned participants to interventions

(no EHEALTH-specific subitems under CONSORT item 10)

Yes, this should be included   It must be specified in the methodology section as to the participants were recruited to different group randomly through a programming protocol or any other protocol.   see above 8 a   No.

11a) If done, who was blinded after assignment to interventions (for example, participants, care providers, those assessing outcomes) and how

NPT: Whether or not administering co-interventions were blinded to group assignment

11a-i) Specify who was blinded, and who wasn't

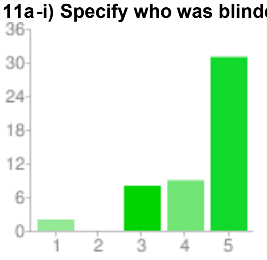

subitem not at all importantessential

1 -subitem not at all important	**2**	4%
2	**0**	0%
3	**8**	15%
4	**9**	16%
5 -essential	**31**	56%

Comment on subitem 11a-i)

Yes. FYI - I've seen growing numbers of journals ask for the work "masking" since blinding is purportedly pejorative. I would also ask that if an assessment of the effectiveness of blinding was performed, that it be provided. (patients often mention their tx assigment to evaluators - it is important to know if that was monitored and if so, how it was managed when it occured). Essential to allow for future comparisons across studies and allow the possibility of being part of meta-analyses Essential Any effects of "non-blinding" on the possible outcomes of the study, should be discussed or at le ...

11a-ii) Discuss e.g., whether participants knew which intervention was the "intervention of interest" and which one was the "comparator"

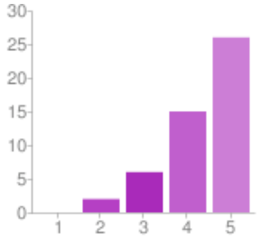

subitem not at all importantessential

1 -subitem not at all important	**0**	0%
2	**2**	4%
3	**6**	11%
4	**15**	27%
5 -essential	**26**	47%

Comment on subitem 11a-ii)

This is the same as a face-to-face trial. This is an important issue. But it is setting a higher level for ehealth than other areas. Essential to allow for future comparisons across studies and allow the possibility of being part of meta-analyses Essential !!! Challenges on developing "sham" e-interventions, "placebo" e-intervention or the "comparator" e-intervention (if appropriate) should be discussed. Now it involves skills as to generate a program which wont let the participants guess. mention in the methodology section. This could be associated to a form of "blinding" Unlike medication, when t ...

Add a subitem under CONSORT item 11a

\- No.

11b) If relevant, description of the similarity of interventions

(this item is usually not relevant for ehealth trials as it refers to similarity of a placebo or sham intervention to a active medication/intervention)

(no EHEALTH-specific subitems under CONSORT item 11b)

Surely it is relevant - eg. were the intervention and comparitor of similar intensity? did they require the same level of engagement (reading just information versus writing replies)? however, it could ofcourse be the case, that patients use other existing forums/bulletin boards/information/wikis on the Internet if they are allocated to the control group etc. This could mimic components of the intervention and consequently could bias your results. Participants should be questioned on this in order to take this into account when interpreting/analysing the results. If more studies are reported ...

12a) Statistical methods used to compare groups for primary and secondary outcomes

NPT: When applicable, details of whether and how the clustering by care providers or centers was addressed

12a-i) Imputation techniques to deal with attrition / missing values

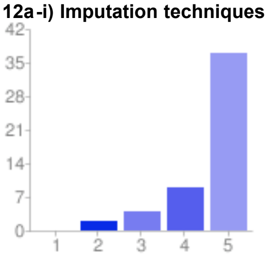

subitem not at all importantessential

1 -subitem not at all important	**0**	0%
2	**2**	4%
3	**4**	7%
4	**9**	16%
5 -essential	**37**	67%

Comment on subitem 12a-i)

Essential to allow for future comparisons across studies and allow the possibility of being part of meta-analyses Important and routine If it's not recommended for other types of RCTs (even if less common) I don't know if eHealth authors should be the first to be singled out or burdened with this as a requirement. Very important and must be properly tabulated in the results section. I suggest this should contain an explicit admonition: "Do not use the term 'intent to treat' unless all initially-enrolled subjects are included in the analysis". (I've seen JMIR papers that failed this.) And then sp ...

Add a subitem under CONSORT item 12a

In the case of clustered randomized trials and repeated measurements, reseracher should be required to conduct multilevel analysis. It is a too common statistical mistake to conduct single-level analysis in cases where there are two or more levels of data. For example, time-level data which are repeatedly measured and person-level data which are measured on one occasion (typically baseline) in a repeated measurements study design. Otherwise, one runs the risk of getting entirely wrong results and drawing wrong conclusions. Include statistical procedures used and why they were chosen - No.

12b) Methods for additional analyses, such as subgroup analyses and adjusted analyses

(no EHEALTH-specific subitems under CONSORT item 12b)

Yes, definitely If Applicable. No. Subgroup analysis should be carefully evaluated, specially when originated from post-hoc analysis. In general they should be avoided.

X26) (not a CONSORT item)

X26-i) Comment on ethics committee approval

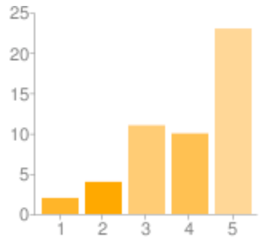

subitem not at all importantessential

1 -subitem not at all important	**2**	4%
2	**4**	7%
3	**11**	20%
4	**10**	18%
5 -essential	**23**	42%

Comment on subitem X26-i)

Most (all?) journals require a statement that for human research IRB approaval was required. It seems appropriate that this is included as a fundamental aspect of research. Essential to allow for future comparisons across studies and allow the possibility of being part of meta-analyses Issues such as Licenses, Intellectual properties, Costs and/or Price(s), Trademarks, Patents, Open- or closed-access of the solution(s) etc. should be provided. Where and when obtained, any adverse events. Between Acknowledgement and Conflict of Interest section.Not needed generally. not sure I understand. Why comm ...

x26-ii) Outline informed consent procedures

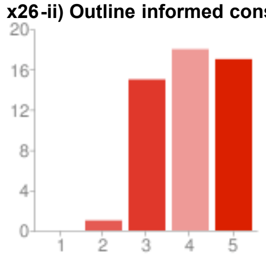

subitem not at all importantessential

1 -subitem not at all important	**0**	0%
2	**1**	2%
3	**15**	27%
4	**18**	33%
5 -essential	**17**	31%

Comment on subitem X26-ii)

An outline of the informed consent is welcome, however, translating and publishing the informed consent is not very important. Essential to allow for future comparisons across studies and allow the possibility of being part of meta-analyses Methods for authentication of the person(s) who signs the informed consent should be discussed. A part in the informed consent should be dedicated to the rights and intellectual properties of the solution developers that should be met by participants. Can be represented as a screen shot. Good point. see above This could be combined with a more complete descr ...

X26-iii) Safety and security procedures

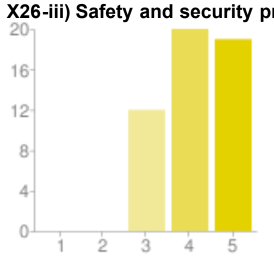

subitem not at all importantessential

1 -subitem not at all important	**0**	0%
2	**0**	0%
3	**12**	22%
4	**20**	36%
5 -essential	**19**	35%

Comment on subitem X26-iii)

Important to know how privacy was guaranteed, because patients have concerns about this. Essential to allow for future comparisons across studies and allow the possibility of being part of meta-analyses If applicable Any specific precautions for participants in special cases should be mentioned; for example the children-rating of the materials and/or contents, the orientation to adults, offensiveness of the material etc. Detection of harm - if human intervention is require or occurs, how quickly it occurs and the outcome of the harm. Appropriate Section. May be after the discussion. Good point. s ...

Add a subitem under item X26

Considering the fact that most ehealth trial collect participant data online, and different database systems / programming languages are available - all with their unique merits and possible security problems - it may be desirable to inform on which database systems / programming languages have been used (both for webbased interventions and webbased datacollection), and how the security measures for the data(bases) were implemented. - No.

RESULTS

13a) For each group, the numbers of participants who were randomly assigned, received intended treatment, and were analysed for the primary outcome

NPT: The number of care providers or centers performing the intervention in each group and the number of patients treated by each care provider in each center

(no EHEALTH-specific subitems under CONSORT item 13a)

Essential to allow for future comparisons across studies and allow the possibility of being part of meta-analyses Any specific method for analysis should be mentioned. For example "intention to treat" etc. Via a Flow Chart or a pictorial representation. No. Number of people *in each analysis*. This is frequently unclear and the number of people in each analysis is essential for calculating an effect for meta-analysis. However, this isn't unique to e-health.

13b) For each group, losses and exclusions after randomisation, together with reasons

13b-i) Attrition diagram

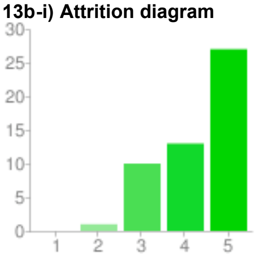

subitem not at all importantessential

1 -subitem not at all important	**0**	0%
2	**1**	2%
3	**10**	18%
4	**13**	24%
5 -essential	**27**	49%

Comment on subitem 13b-i)

An interesting contrast to drug trials - where self-report or pill count is all that is often done to evaluate 'adherence' with treatment. I would distinguish attrition from intervention from lost-to-follow-up from assessment. Specify both drop-out and non-use attrition separately We did not have any way to collect reasons for discontinuing participation - as we had no contact with our participants other than email/online intervention. We had no IRB permission to request details of discontinuing participation. There are many cases in which reasons for attrition would be available. Essential to ...

Add a subitem under CONSORT item 13b

Describe whether there was differential attrition (i.e., a differences in attrition between the two groups) and how was dealt with this. It is covered above under methods, but I would have use data here. It is really important to report a clear metric for use, separate from attrition. Someone who logs into a site every day over 12 weeks has a different dose from someone who logs in once every week. And I suspect this will be important to systematic reviewers in the future. Details of why people dropped out would be helpful to determine what didn't work and how this may have impacted on the ...

14a) Dates defining the periods of recruitment and follow-up

14a-i) Indicate if critical “secular events” [1] fell into the study period

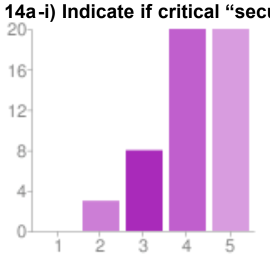

subitem not at all importantessential

1 -subitem not at all important	**0**	0%
2	**3**	5%
3	**8**	15%
4	**20**	36%
5 -essential	**20**	36%

Comment on subitem 14a-i)

Essential to allow for future comparisons across studies and allow the possibility of being part of meta-analyses Japan is a recent example. Experiment wise, in the methodology section. only month and year would be enough. it's a co-intervention related to 3b-i and 5iii Good point. However, if these events have been described in the "Method" section, it is probably a redundant to mention it again in the "Results" section here. I can see how this is important, but I'm not sure it's essential.

Add a subitem under CONSORT item 14a

Would this include events like Hurricane Katrina or 9/11, in which cases people would rather watch television that use a web-based intervention. - No.

14b) Why the trial ended or was stopped (early)

(no EHEALTH-specific subitems under CONSORT item 14b)

this could also be the case in ehealth trials. So I would not omit this item from this checklist. Yes, this is would be important to include in case anyone else may want to try and replicate the study Reason should be quoted in the discussion. No. EHEALTH-specific additions here: Secular events, political regime change in the instance of countries choosing to withdraw from the Internet.

15) A table showing baseline demographic and clinical characteristics for each group

NPT: When applicable, a description of care providers (case volume, qualification, expertise, etc.) and centers (volume) in each group

15-i) Report demographics associated with digital divide issues

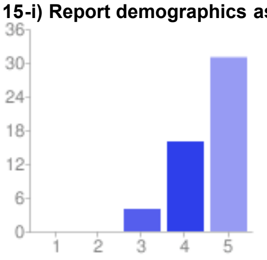

subitem not at all importantessential

1 -subitem not at all important	**0**	0%
2	**0**	0%
3	**4**	7%
4	**16**	29%
5 -essential	**31**	56%

Comment on subitem 15-i)

If known Essential to allow for future comparisons across studies and allow the possibility of being part of meta-analyses As available What rapid computer literacy assessment tool can be recommended? Always beneficial and illustrative. Good point. table on demographics is important but unsure about digitcal divide Clearly relevant, but perhaps not in this guideline. Seems to be related to standardised outcome sets?

Add a subitem under CONSORT item 15

See my comments under item 12a. In case of including multilevel analysis as a necessary statistical procedure as required by the study design, separate correlation matrices for variables at the different levels should be included - at least in multilevel regression analysis, latent growth curves and most probably multilevel structural equation models. Maybe some additional guidelines on some standard ways of tabulating the demographic data would be very useful - No.

16) For each group, number of participants (denominator) included in each analysis and whether the analysis was by original assigned groups

16-i) Report multiple "denominators" and provide definitions

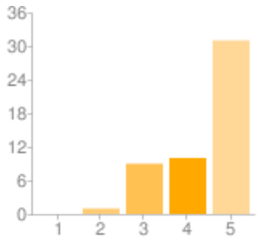

subitem not at all importantessential

1 -subitem not at all important	**0**	0%
2	**1**	2%
3	**9**	16%
4	**10**	18%
5 -essential	**31**	56%

Comment on subitem 16-i)

While these multiple outcomes are of interest surely the primary measure for an RCT should be ITT? Essential to allow for future comparisons across studies and allow the possibility of being part of meta-analyses Essential ! Already outlined in the Flow chart. Separately mentioning these things would increase the length of the paper. Somethings should be indirectly inferred and interpreted from the tables and figures which should be briefly described in the discussion section. Good point. N eligible to be exposed to intervention should also be indicated. This is a good principle, but too vaguely ...

16-ii) Primary analysis should be intent-to-treat

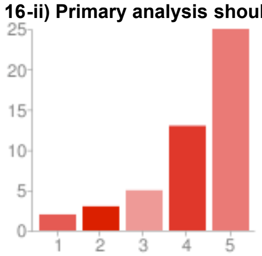

subitem not at all importantessential

1 -subitem not at all important	**2**	4%
2	**3**	5%
3	**5**	9%
4	**13**	24%
5 -essential	**25**	45%

Comment on subitem 16-ii)

Important to distinguish between effectiveness analyses (i.e. analysis of the whole sample) and efficacy analyses (i.e. sub-group analyses) which in the latter case is no longer a randomized sample. This should be made explicit in all papers. However, the intent-to-treat principle has its advantages, but also disadvantages. It seems to me that the statistical and methodological scientific community is becoming more and more pro imputation techniques because of the disadvantages associated with the ITT principle. Thus, I don't think that the primary analysis should or must be intent-to-treat ...

Add a subitem under CONSORT item 16

\- No.

17a) For each primary and secondary outcome, results for each group, and the estimated effect size and its precision (such as 95% confidence interval)

17a-i) Presentation of process outcomes such as metrics of use and intensity of use

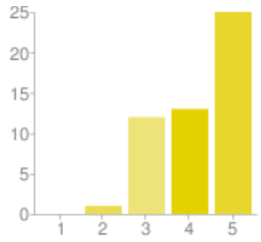

subitem not at all important essential

1 -subitem not at all important	**0**	0%
2	**1**	2%
3	**12**	22%
4	**13**	24%
5 -essential	**25**	45%

Comment on subitem 17a-i)

Usage metrics are critically important, but "average session length" is problematic when users access online interventions outside the laboratory. Even when timeouts are used, how do we know that users are attending to the intervention when the clock is ticking? Separate analyses should be run on self-reported and objective usage data as these are differently correlated with e.g. self-reported questionnaires. Self-reported usage data correlate more highly with other self-reported questionnaire data than more objective usage data. Yes - seems to address one of my comments above. Essential to al ...

Add a subitem under CONSORT item 17a

- No.

17b) For binary outcomes, presentation of both absolute and relative effect sizes is recommended

(no EHEALTH-specific subitems under CONSORT item 17b)

Essential to allow for future comparisons across studies and allow the possibility of being part of meta-analyses Have to be incorporated as per the case.Most useful in case of exact replication otherwise absolute effect sizes would be sufficient. No. Once again, the real issue is the robustness of the findings to variations in analytical methods. Emphasis to use NNT and NNH- number needed to treat and number needed to harm whem possible

18) Results of any other analyses performed, including subgroup analyses and adjusted analyses, distinguishing pre-specified from exploratory

18-i) Subgroup analysis of comparing only users

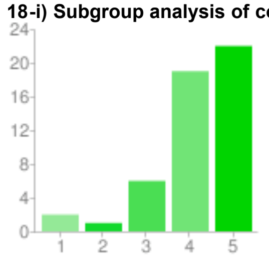

subitem not at all important essential

1 -subitem not at all important	**2**	4%
2	**1**	2%
3	**6**	11%
4	**19**	35%
5 -essential	**22**	40%

Comment on subitem 18-i)

I'm not sure subgroup analysis is always "self-selected" and they are sometimes the primary hypothesis. Essential to allow for future comparisons across studies and allow the possibility of being part of meta-analyses Important, if such analyses were performed Appropriately done. pragmatic RCTs should reflect real world situations - 'only users' analysis is of limited use and does not reflect how the intervention would perform outside the trial. Good point. If a subgroup analysis of comparing only users is reported, proper analytic procedures should be included in "Method" section too, They shou ...

Add a subitem under CONSORT item 18

- No.

19) All important harms or unintended effects in each group

(for specific guidance see CONSORT for harms)

19-i) Include privacy breaches, technical problems

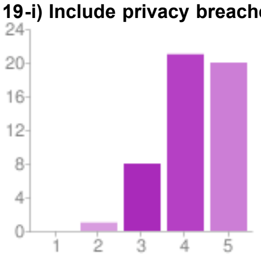

subitem not at all importantessential

1 -subitem not at all important	**0**	0%
2	**1**	2%
3	**8**	15%
4	**21**	38%
5 -essential	**20**	36%

Comment on subitem 19-i)

I would put technical issue / privacy breaches in the IRB field - but report unitended consequences. Might clarify level of harm - sounds like adding privacy breaches to adverse events reporting, which is important. Essential to allow for future comparisons across studies and allow the possibility of being part of meta-analyses If applicable If they are of magnitude higher than the one fixed by the regulating agency. related to 3b-i; 5iii and 14a-i Any adverse events should be reported. It is important especially when people using communication technology may be in a greater risks of any assault ...

19-ii) Include qualitative feedback from participants or observations from staff/researchers

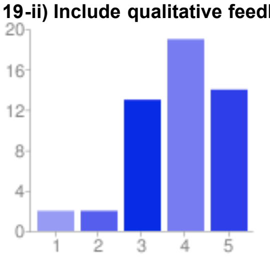

subitem not at all importantessential

1 -subitem not at all important	**2**	4%
2	**2**	4%
3	**13**	24%
4	**19**	35%
5 -essential	**14**	25%

Comment on subitem 19-ii)

It is time to slice the salami! A good quality qualitative paper is justified but a paragraph in the primary paper seems unlikely to adequately address the issues. With space limitations, this report would be limited in scope to 1 or 2 sentences. I do not think it should be required. Essential to allow for future comparisons across studies and allow the possibility of being part of meta-analyses Hmm . . . This really depends on the extent to which the qual data affected analysis and interpretation. In order to improve the website and make it more user friendly. It is very important as descriptiv ...

Add a subitem under CONSORT item 19

Maybe some guidelines about how to quantify qualitative feedback to make it more useful to others who may want to replicate the study - No.

DISCUSSION

22) Interpretation consistent with results, balancing benefits and harms, and considering other relevant evidence

NPT: In addition, take into account the choice of the comparator, lack of or partial blinding, and unequal expertise of care providers or centers in each group

22-i) Restate study questions and summarize the answers suggested by the data [2], starting with primary outcomes and process outcomes (use)

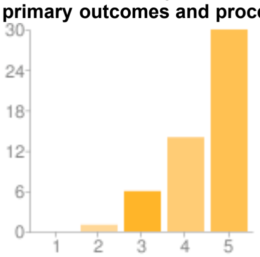

1 -subitem not at all important	**0**	0%
2	**1**	2%
3	**6**	11%
4	**14**	25%
5 -essential	**30**	55%

Comment on subitem 22-i)

I would also like to stress the importance of utilizing RCTs to conduct process research which includes far more than just usage. Process research could for example include data on mediation and moderation effects. How do participatns develop on theoretically relevant variables during the intervention? And how do these variables relate to primary outcomes? Are there any participant characteristics that interact with intervention assignment? Etc. Essential to allow for future comparisons across studies and allow the possibility of being part of meta-analyses Essential Discussion part should be i ...

22-ii) Highlight unanswered new questions, suggest future research [2]

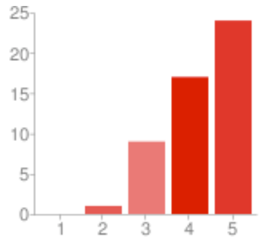

subitem not at all importantessential

1 -subitem not at all important	**0**	0%
2	**1**	2%
3	**9**	16%
4	**17**	31%
5 -essential	**24**	44%

Comment on subitem 22-ii)

Essential to allow for future comparisons across studies and allow the possibility of being part of meta-analyses Essential I think mentioning the reproducibility of the intervention in other platforms is an appropriate suggestion for future research. This will provide direction and the the researcher's wisdom and perspective to the potential new researcher. Good point. Is this not redundant? This doesn't add much to the current version of CONSORT.

Add a subitem under CONSORT item 22

- It would be very useful if researchers could emphasize and elaborate on their lessons learned in the "design" and "implementation" of the intervention based on both primary and process outcome findings as well as unintended outcome findings. This information would be not only important for readers to see the actual picture of the intervention effects but also very beneficial to future researchers and developers so that the same lessons won't need to be learned again.

20) Trial limitations, addressing sources of potential bias, imprecision, and, if relevant, multiplicity of analyses

20-i) Typical limitations in ehealth trials

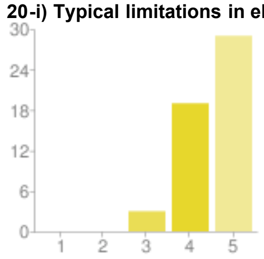

subitem not at all importantessential

1 -subitem not at all important	**0**	0%
2	**0**	0%
3	**3**	5%
4	**19**	35%
5 -essential	**29**	53%

Comment on subitem 20-i)

Essential to allow for future comparisons across studies and allow the possibility of being part of meta-analyses Essential Measures taken by investigators to lessen the amount and limit the sources of biases should be discussed. Last section should focus on the limitations and delimitation's. Good point. Also redundant? However nive to have in a list as many reporting e-health stuies do not have a substantial social science research methods education More thought is needed about how this would add to the current version of CONSORT.

Add a subitem under CONSORT item 20

Use and differential attrition deserve extra attention in eHealth trials. - No.

21) Generalisability (external validity, applicability) of the trial findings

NPT: External validity of the trial findings according to the intervention, comparators, patients, and care providers or centers involved in the trial

21-i) Generalizability to other populations

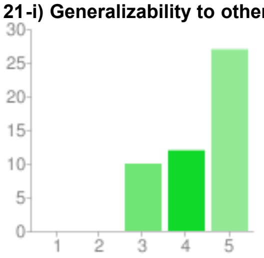

subitem not at all importantessential

1 -subitem not at all important	**0**	0%
2	**0**	0%
3	**10**	18%
4	**12**	22%
5 -essential	**27**	49%

Comment on subitem 21-i)

Implementation of successful E-health interventions will be crucial in the following years Essential Any assumption and pre-requisite made by the investigators that seems to have a positive/negative effects on generalizability of findings should be discussed in this part. And avoid over-generalizing the implications of the study (a danger with eHealth trials). depends from a study to other. Generalization again will be difficult. or ex If a particular study is designed keeping in mind the study habits and achievement of Indian students it cannot be generalized beyond the territories of the the n ...

21-ii) Discuss if there were elements in the RCT that would be different in a routine application setting

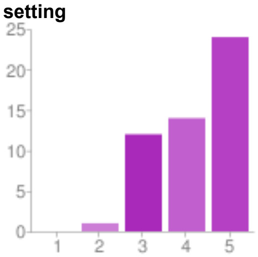

subitem not at all importantessential

1 -subitem not at all important	**0**	0%
2	**1**	2%
3	**12**	22%
4	**14**	25%
5 -essential	**24**	44%

Comment on subitem 21-ii)

Important!!! Useful for readers to know how to implement this intervention in daily practice. Essential to allow for future comparisons across studies and allow the possibility of being part of meta-analyses Only of applicable Yes, the alternative mechanisms should be rightfully mentioned. Good point. same as above Plus information about program availability, restrictions and costs. I think this is very relevant, but procedures should be specified in

the methods section, not the discussion.

Add a subitem under CONSORT item 21

-

OTHER INFORMATION

23) Registration number and name of trial registry

(no EHEALTH-specific subitems under CONSORT item 23)

Yes, this would be important to include. If all this is in a database then interested parties may be able to search and find similar studies, etc. Any registration number of the developed solutions can be mentioned too. Optional, depend upon the researcher. Mostly a name is provided like STUDENT WELL BEING ENHANCEMENT PROGRAM, Depression prevention program, Happier program etc. No. Agreed.

24) Where the full trial protocol can be accessed, if available

(no EHEALTH-specific subitems under CONSORT item 24)

An online database would be good. Contact Authors.Best person to tell about the protocols used. No. Agreed.

25) Sources of funding and other support (such as supply of drugs), role of funders

(no EHEALTH-specific subitems under CONSORT item 25)

Yes, definitely, as part of sponsorships and conflict of interest disclosures Sources of funding and other support from businesses are so important in EHEALTH-specific RCTs. Whatever the case may be (in acknowledgements) No. Agreed.

X27) (not a CONSORT item)

X27-i) State the “relation of the study team towards the system being evaluated”

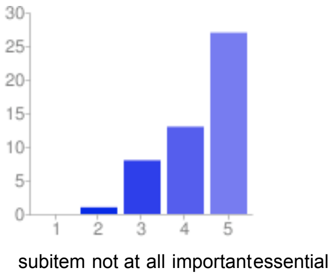

1 -subitem not at all important	**0**	0%
2	**1**	2%
3	**8**	15%
4	**13**	24%
5 -essential	**27**	49%

Comment on subitem X27-i)

As per the ISRII discussion - this is generally not explicity stated in face-to-face trials where it is a similar problem. Essential to allow for future comparisons across studies and allow the possibility of being part of meta-analyses As applicable. Vary from journal to journal. Good point. Absolutely right.

Add a subitem under item X27

Maybe some more detail about what is meant by "system being evaluated." A list of key words would be good for purposes of future online database searching. - No.

Last question

Do you want to become involved in the writing committee working on the elaboration document? If yes, please provide the subitems you wish to elaborate on

5-ii - developing a framework for a logical/process model 6a-i, 7a-i, 12a-i, 16-ii, X26 use: 5-ix, 5-xi, 6a-ii; differential attrition: 12a, 13b Possibly 4a-ii, 5viii, 5x, insertion of addition item in ...

Number of daily responses

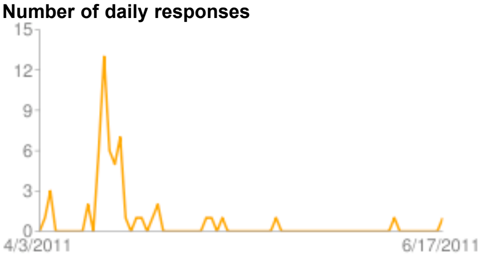

Supplement: Supplementary file 2 [file jmir_v13i4e126_app2.pdf]
